# A KH-Domain RNA-Binding Protein Interacts with FIERY2/CTD Phosphatase-Like 1 and Splicing Factors and Is Important for Pre-mRNA Splicing in Arabidopsis

**DOI:** 10.1371/journal.pgen.1003875

**Published:** 2013-10-17

**Authors:** Tao Chen, Peng Cui, Hao Chen, Shahjahan Ali, Shoudong Zhang, Liming Xiong

**Affiliations:** Division of Biological and Environmental Sciences and Engineering, King Abdullah University of Science and Technology (KAUST), Thuwal, Saudi Arabia; University of California Riverside, United States of America

## Abstract

Eukaryotic genomes encode hundreds of RNA-binding proteins, yet the functions of most of these proteins are unknown. In a genetic study of stress signal transduction in Arabidopsis, we identified a K homology (KH)-domain RNA-binding protein, HOS5 (High Osmotic Stress Gene Expression 5), as required for stress gene regulation and stress tolerance. HOS5 was found to interact with FIERY2/RNA polymerase II (RNAP II) carboxyl terminal domain (CTD) phosphatase-like 1 (FRY2/CPL1) both in vitro and in vivo. This interaction is mediated by the first double-stranded RNA-binding domain of FRY2/CPL1 and the KH domains of HOS5. Interestingly, both HOS5 and FRY2/CPL1 also interact with two novel serine-arginine (SR)-rich splicing factors, RS40 and RS41, in nuclear speckles. Importantly, FRY2/CPL1 is required for the recruitment of HOS5. In *fry2* mutants, HOS5 failed to be localized in nuclear speckles but was found mainly in the nucleoplasm. *hos5* mutants were impaired in mRNA export and accumulated a significant amount of mRNA in the nuclei, particularly under salt stress conditions. Arabidopsis mutants of all these genes exhibit similar stress-sensitive phenotypes. RNA-seq analyses of these mutants detected significant intron retention in many stress-related genes under salt stress but not under normal conditions. Our study not only identified several novel regulators of pre-mRNA processing as important for plant stress response but also suggested that, in addition to RNAP II CTD that is a well-recognized platform for the recruitment of mRNA processing factors, FRY2/CPL1 may also recruit specific factors to regulate the co-transcriptional processing of certain transcripts to deal with environmental challenges.

## Author Summary

Pre-mRNA processing, including 5′ capping, splicing, and 3′ polyadenylation, is critical for gene expression and is closely coupled with transcription. Phosphorylated carboxyl terminal domain (CTD) of RNA Polymerase II (RNAP II) serves as a platform for the recruitment of pre-mRNA processing factors, yet other components involved in the recruitment are less known. In a genetic study of stress signal transduction in Arabidopsis, we isolated a KH-domain RNA-binding protein HOS5 that plays important roles in stress gene regulation and stress tolerance. We found that HOS5 interacts with FIERY2/CTD phosphatase-like 1 (FRY2/CPL1) and they both also interact with two novel splicing factors, RS40 and RS41, in nuclear speckles. In *fry2* mutants, HOS5 was unable to be recruited to nuclear speckles but rather was mainly localized in the nucleoplasm. Mutants in these genes have similar stress-sensitive phenotypes. Transcriptome analyses identified significant intron retention in many stress-related genes in these mutants under salt stress conditions. Our study reveals that, in addition to RNAP II, the CTD phosphatase may also recruit specific splicing factors and RNA binding proteins to regulate the co-transcriptional processing of certain transcripts to deal with environmental stresses.

## Introduction

Pre-mRNA processing, including 5′ capping, splicing and 3′ end formation, is highly regulated and often coupled with transcription to increase its accuracy and efficiency. The carboxyl terminal domain (CTD) of Rpb1, the largest subunit of RNA polymerase II (RNAP II), serves as a platform for the recruitment and assembly of these processing factors [1]. RNAP II CTD consists of tandem YSPTSPS heptad repeats that can be modified most frequently by phosphorylation/dephosphorylation. The combination of different modifications among the heptapeptide repeats defines the so-called ‘CTD codes’ that correlate with the progression of the transcription cycle [2] and also regulate transcript processing [3]. CTD phosphorylation results in differential recruitment of RNA processing factors to the nascent transcripts. For instance, phosphorylated Ser5 recruits 5′ capping enzymes whereas phosphorylated Ser2 recruits 3′ end processing factors [4], [5], although the relatedness of CTD phosphorylation to splicing is more complicated, perhaps partly due to the complexity of the splicing machinery itself.

Pre-mRNA splicing is an important step in mRNA maturation in eukaryotes. The accurate recognition and excision of introns are vital and require the coordinated function of a large number of proteins. Splicing takes place in the spliceosome, a large RNA-protein complex that includes 5 snRNAs and approximately 180 proteins [6]. The complexity of the spliceosome provides ample opportunities for regulation and one major output of the regulation is the alternative splicing (AS) of pre-mRNA. AS also controls the gene expression level by producing transcript variants that are degraded by the nonsense-mediated decay (NMD) pathway [7].

There are several common types of AS including intron retention, exon skipping, and alternative 5′ splice site or 3′ splice site selection. Among these types, intron retention has been shown to be the most frequent type of AS in Arabidopsis and other plants [8]. For instance, in Arabidopsis, these four types of AS respectively account for 24.21, 2.73, 7.55, and 15.46% of the total AS events and the remaining AS events are a combination of these four types [9], [10]. Alternative splicing could be regulated by plant developmental stages as well as by many environmental conditions such as biotic or abiotic stresses [11], [12]. Likely, these internal and external signals affect splicing via their regulation of specific splicing factors.

One important group of splicing factors is the Serine/Arginine-rich protein group (SR proteins). These SR proteins are also involved in mRNA nuclear export, mRNA stability control, and genome maintenance [13]–[16]. SR proteins possess one or two RNA recognition motifs (RRM) at the N-terminus and one Ser/Arg-rich region at the C-terminus [17]. There are 19 SR genes in the Arabidopsis genome. Interestingly, these genes can also be alternatively spliced in different tissues or at different development stages, and under hormone or stress treatments [18]. For example, *SR30* has been shown to have intron retention in the *atprmt5* mutant [19]; alternative splicing of *RS40* was detected in the *upf1*-5 and *upf3*-1 mutants or after CHX treatment [7]; and alternative splicing of SR-related gene *SR45a* has been observed under high light, salt, heat, and dehydration stress conditions [20], [21].

To understand gene regulation mechanisms under osmotic stress, we screened for Arabidopsis mutants with abnormal expression of the stress-inducible reporter gene *RD29A*::*LUC* (*RD29A* promoter driven the firefly *LUCIFERASE* gene) [22]. One mutant, *hos5-1 (high osmotic stress gene expression 5-1)*, that showed higher levels of *LUC* expression than the wild type under NaCl or the phytohormone abscisic acid (ABA) treatment, was isolated previously [23], although the causal gene had not been identified. Here, we conducted map-based cloning and found that *HOS5* encodes a KH-domain RNA-binding protein that was localized in the nuclear speckles. We also found that HOS5 interacts with two SR proteins and FIERY2/RNAP II CTD phosphatase-like 1 (FRY2/CPL1). Interestingly, the subnuclear localization of HOS5 depends on FRY2/CPL1. In *fry2-1* mutants, HOS5 was no longer localized in nuclear speckles but rather mainly in the nucleoplasm. FRY2/CPL1 also interacts with the two SR splicing factors and all these mutants exhibited similar ABA- and salt-sensitive phenotypes as *hos5*. RNA-seq analyses revealed that many transcripts were misspliced with one or more introns retained in the mutants under salt stress conditions. The current study identified HOS5 as a novel factor involved in splicing and stress response. We proposed that FIERY2/CTD phosphatase-like 1 (FRY2/CPL1), like CTD itself, might help to recruit specific RNA processing factors for co-transcriptional processing of nascent transcripts to deal with environmental stresses.

## Results

### HOS5 encodes a KH-domain RNA-binding protein

We previously isolated the *hos5-1* mutant by screening for altered regulation of stress-responsive genes using the *RD29A-LUC* reporter [23]. As shown in our previous study and in Supplementary Figure S1 (Figure S1), compared with the wild-type *RD29A-LUC* line (C24 background, hereafter referred to as the wild type or C24), *hos5-1* mutant seedlings exhibited brighter luminescence after treatment with 100 µM ABA (Figure S1B) or 300 mM NaCl (Figure S1C). Quantitative RT-PCR confirmed the high expression level of *LUC* in the *hos5*-*1* mutant (Figure S1D). Furthermore, *hos5-1* seed germination and seedling growth were more sensitive to ABA and salt stress than were the wild type (Figures S1E to S1I). Under NaCl treatments, the color of the leaves of *hos5-1* was more yellowish than that of the wild-type seedlings, supporting the notion that the *hos5-1* whole seedling was more sensitive to salt stress.

**Figure 1:**
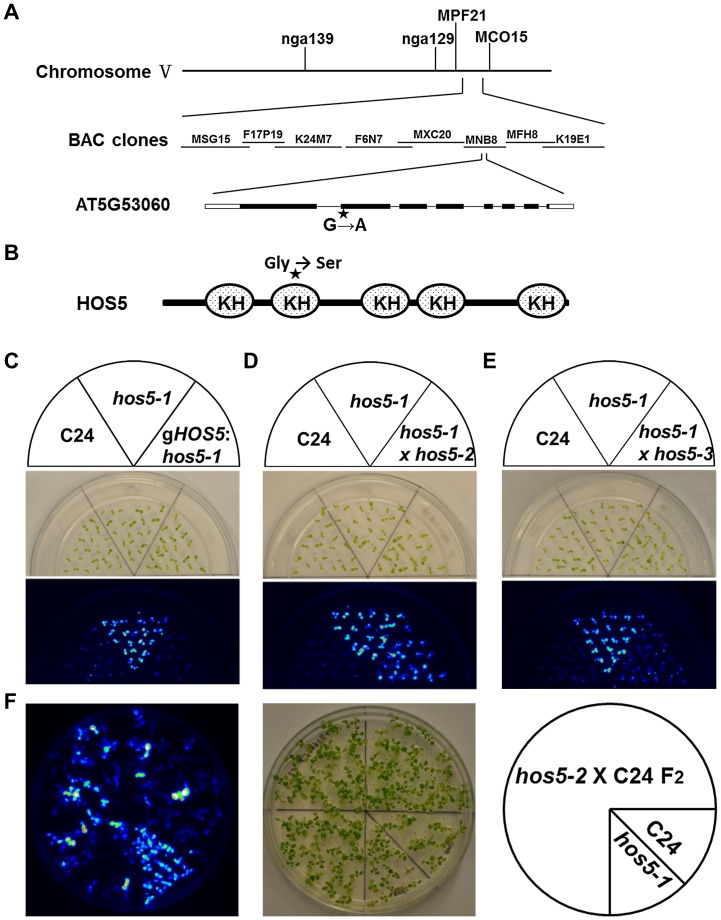
Positional cloning of the *HOS5* gene and complementation of the *hos5-1* mutant. (A) *HOS5* is localized on Chromosome V. Schematic structure of the *HOS5* gene. Black boxes, exons; white boxes, UTRs; lines, introns; star, the *hos5*-*1* mutation site. (B) Structure of HOS5 protein and position of *hos5-1* mutation. (C–F) Complementation of the *hos5-1* phenotype by the *HOS5* gene (C), allelism testing (D–E), and segregation of a F_2_ population (F). Ten-day-old seedlings grown in a ½ MS (Murashige and Skoog) agar medium were treated with 300 mM NaCl for 3 hr before imaging with a CCD camera. Shown are morphology of the seedlings and luminescence images. C24, the wild type (with *RD29A-LUC* reporter); *gHOS5:hos5-1, hos5-1* plants expressing the wild-type *HOS5* genomic DNA; *hos5*-*1*×*hos5*-*2*, F_1_ progeny of a cross between the two mutants; *hos5*-*1*×*hos5*-*3*, F_1_ progeny of a cross between the two mutants; *hos5-2*×C24 F_2_, segregated population of the F_2_ generation of a cross between *hos5-2* and wild type C24.

To map the *HOS5* gene, we generated a mapping population from a cross between *hos5-1* and the Columbia-0 (Col-0) ecotype. Genetic mapping delimited the *HOS5* locus to the BAC clone MNB8 on Chromosome 5. The genomic DNA in this region was amplified from the wild-type and *hos5-1* mutant plants and sequenced. A single G to A mutation in the second exon of the At5g53060 gene was found. This gene encodes a KH-domain protein (Figure 1A). The mutation changed a codon of GGT to AGT and resulted in a substitution of Gly at position 233 by Ser in the corresponding protein in the *hos5-1* mutant (Figure 1B).

The At5g53060 gene, which was recently identified as RCF3 [24], encodes a protein with five KH domains. The three-dimensional structure of the protein predicted by Phyre2 [25] shows that each KH domain is a α–helix (Figure S2A). The mutation in *hos5-1* occurred in the second KH domain. A cladogram analysis of this second KH domain in KH domain-containing proteins in Arabidopsis showed that it has the most similarity to the one in BTR1, a positive strand virus RNA-binding protein [26] (Figure S2B). We found that the most conserved consensus sequence in the KH domain, which is 60 amino acids long and forms a hydrophobic α–helix, was VIGXXGXXI. In addition, glycine at position 233 is the most conserved residue in this domain and a mutation at this site, as occurred in *hos5-1*, may lead to changes in RNA recognition or binding or in protein-protein interaction (Figure S2C).

To confirm that At5g53060 is the *HOS5* gene, we cloned its genomic DNA, including the coding sequence and the promoter, into the binary vector pMDC123 and introduced it into the *hos5-1* mutant by *Agrobacterium*-mediated transformation. The T_3_ generation of the transgenic plants was then analyzed for luminescence after NaCl treatment (Figure 1C). The brighter luminescence phenotype previously observed in *hos5-1* disappeared in the plants transformed with the At5g53060 genomic DNA and the luminescence level became similar to that in the wild-type plants, suggesting that this gene complemented the *hos5*-1 mutation and was indeed *HOS5*. We also obtained two T-DNA insertion lines, SALK-095666 and SALK-143161, which were subsequently named *hos5-2* and *hos5-3*, respectively (Figure S3A). Examination of the transcript levels in these two mutants confirmed that they were null mutants (Figure S3B). The *hos5-1* mutant was next crossed with *hos5-2* and *hos5-3*. The F_1_ seedlings were subjected to luminescence analysis after NaCl treatment (Figures 1D and 1E). Both of the two F_1_ lines had brighter luminescence than that of the wild type but similar to that of *hos5-1*. *hos5-2* was also crossed with the wild-type C24 plant and approximately 19% of the resulting F_2_ seedlings had luminescence as bright as that of *hos5-1* (Figure 1F). These results together indicate that At5g53060 is *HOS5* and that mutations in this gene result in the higher expression of the *RD29A*-*LUC* reporter gene.

We examined whether the other *hos5* mutant allele was also sensitive to stress. The *hos5-2* seedlings were more sensitive to NaCl, with shorter roots and smaller rosette sizes compared with the wild-type Col-0 (referred to as Col-0) plants (Figures S3C and S3D). Compared with Col-0, the germination of *hos5-2* mutant seeds (Figure S3E) as well as root elongation (Figure S3F) was more inhibited by ABA. These results indicate that *hos5-2* is sensitive to NaCl and ABA, similar to *hos5-1*.

### The expression and subcellular localization of HOS5

**Figure 2:**
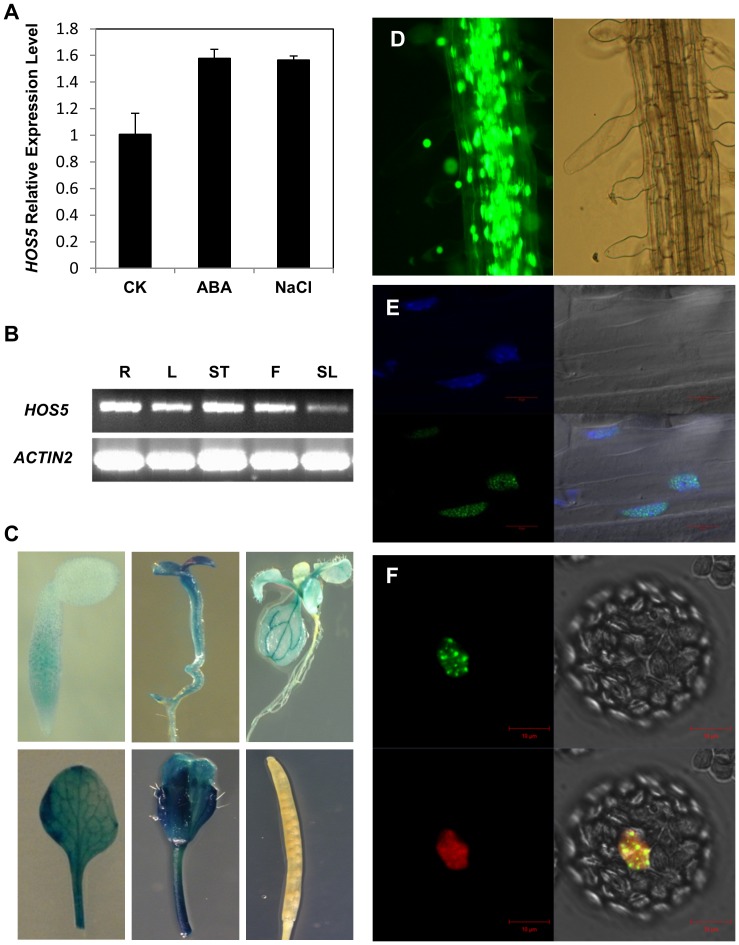
Gene expression pattern of *HOS5*. (A) qPCR analysis of *HOS5* expression. Ten-day old seedlings were treated with 100 µM ABA or 300 NaCl for 3 hr before extracting RNA for the analysis. Error bars represent the standard deviations (*n* = 3). (B) *HOS5* expression level in different parts of plants. R, root; L, leaf; ST, stem; F, flower; SL, silique. *ACT2* was used as the internal control in qPCR assays in (A) and (B). (C) *HOS5* promoter driven *GUS* expression. Transgenic plants expressing *GUS* under control of the *HOS5* promoter were stained with X-Gluc and imaged under a microscope. (D) HOS5-GFP fusion protein is localized in the nucleus. Root cells of HOS5-GFP transgenic plant were imaged under a fluorescence microscope. Left panel, GFP image, right panel, bright field. (E) Subcellular localization of HOS5. Shown are confocal images of root cells expressing HOS5-GFP fusion protein in green (left lower panel) and DAPI staining in blue (left upper panel), and merged image of GFP and DAPI (right lower panel). (F) HOS5 co-localized with CypRS64. HOS5-GFP and CypRS64-RFP fusion protein were co-transformed into Arabidopsis protoplasts and transiently expressed. Images were taken using a confocal microscope 16 h after the transformation. Bars in (E) and (F) indicate the scale.

To determine the regulation of the *HOS5* gene, Col-0 seedlings were treated with 100 µM ABA or 300 mM NaCl for 3 hr and total RNA was extracted and was reverse transcribed to cDNA. We found that the transcript level of *HOS5* was slightly up regulated in response to ABA and salt treatment as determined by quantitative real-time PCR (qPCR) (Figure 2A). *HOS5* was expressed ubiquitously albeit the expression level was lower in siliques. Transgenic plants expressing the *HOS5* promoter fused with the beta-glucuronidase (*GUS*) reporter gene were generated and GUS expression in independent lines was examined. GUS expression patterns (Figure 2C) were similar to those of the RT-PCR results (Figure 2B).

To examine the subcellular localization of the HOS5 protein, stable transgenic Arabidopsis plants expressing HOS5 fused in frame with the green fluorescent protein (GFP) at the C-terminus were obtained. Several independent lines were examined for GFP expression. The GFP signals were detected in the nucleus of root cells by fluorescence microscopy (Figure 2D). This nuclear localization was further confirmed by co-localization of the GFP signal with DAPI signal (Figure 2E). Interestingly, the confocal images showed that HOS5 is localized in nuclear structures very much like nuclear speckles, where many pre-mRNA splicing factors are localized. We therefore decided to investigate this possibility further.

The splicing factor CypRS64 has been used as a marker for pre-mRNA splicing and it is localized in nuclear bodies and splicing speckles [27], [28]. We constructed and co-transformed CypRS64-mRFP into Arabidopsis protoplast with the HOS5-GFP plasmid via PEG-mediated transformation [29]. Sixteen hours after the transformation, fluorescence images were captured using a confocal microscope. While HOS5-GFP was mostly found in the nuclear speckles, CypRS64 was found in the nuclear speckles but also in nucleoplasm, albeit to a much lesser extent (Figure 2F), similar to previously reported [27], [28]. The co-localization of HOS5 and CypRS64 in nuclear speckles implies that HOS5 may play a role in pre-mRNA splicing or processing.

### HOS5 interacts with FRY2/CPL1 and two novel RS splicing factors

**Figure 3:**
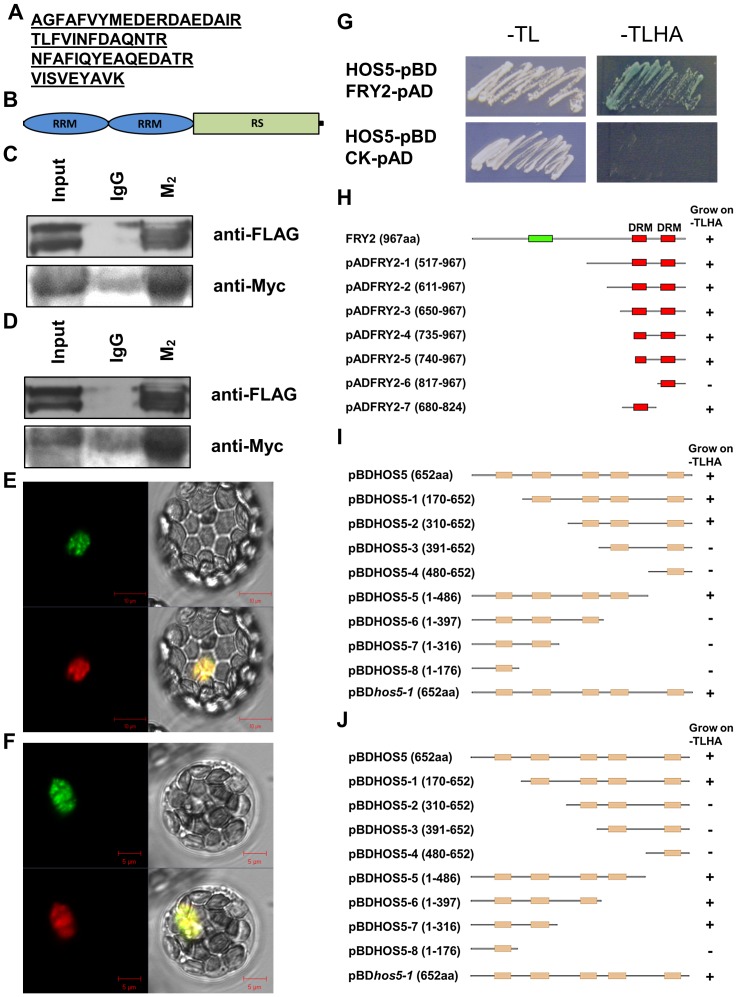
HOS5 interacts with FRY2 and two RS proteins. (A) Four peptide fragments of RS40 and RS41 proteins were identified by mass spectrometry after Co-IP. (B) Structure of RS40 and RS41 proteins. Ellipse, RNA recognition motif (RRM); rectangle, RS domain. (C) to (D) Analysis of in vivo interaction between HOS5, RS40 and RS41. The plasmids for HOS5-FLAG, RS40-Myc and RS41-Myc fusion protein were transiently expressed in tobacco leaves. Proteins were immunoprecipitated from total protein extracts with anti-FLAG M2 beads (IgG used as control) and analyzed by immunoblots with anti-FLAG M2 to detect HOS5-FLAG and anti-Myc antibody to detect RS40 (C) and RS41 (D). (E) to (F) HOS5 co-localized with RS40 and RS41 respectively. Plasmids of HOS5-GFP along with RS40-RFP (E) and RS40-RFP (F) were co-transformed into Arabidopsis protoplasts. Images were taken 16 hr after transformation using a confocal microscope. Clockwise from the upper left panel: HOS5 (green); bright field; RS40 or RS41 (red); merged (yellow). Bars indicate the scales. (G) HOS5 interacts with FRY2 in yeast. HOS5 was cloned into pBD, and FRY2 was cloned into pAD. Empty pAD vector was used as a control. The combinations of plasmids were transformed into yeast and grow on -tryptophan/-leucine (-TL) media for transformation validation and on -tryptophan/-leucine/-histidine/-adenine (-TLHA) media for interaction validation. (H) To (J) Mapping the HOS5-FRY2 interaction and HOS5 dimerization domains. FRY2 and HOS5 deletion constructs were made from both the amino and carboxy termini as indicated. These deletion mutants were then tested for interaction in the yeast two-hybrid system by co-transforming into yeast strain AH109 with the corresponding full-length partner (*FRY2* in pAD or *HOS5* in pBD) and plating on -TL (transformation control) and -TLHA (selection) nutrient media plus X-α-Gal. Seven deletion constructs of FRY2 and eight deletion constructs of HOS5 along with hos5-1 mutant were tested. (H) Mapping the domain of FRY2 that interacts with HOS5. (I) Mapping the domain of HOS5 that interacts with FRY2. (J) Mapping the domain of HOS5 for dimerization.

In order to understand the molecular mechanisms of HOS5 in gene regulation, we investigated whether or not there are any proteins that interact with HOS5. We first conducted a yeast two-hybrid screen using HOS5 as bait. The screen identified two proteins that interact with HOS5. One turned out to be HOS5 itself, suggesting that HOS5 may form a homodimer (data not shown). The second HOS5-interacting protein was FIERY2/RNAP II CTD phosphatase-like 1 (FRY2/CPL1), which was identified in the same genetic screen as *hos5-1* [30]–[32] (Figure 3G). Notably, FRY2/CPL1 mutations conferred an increased expression of stress-responsive genes, a phenotype similar to that of *hos5*. To verify this interaction, we used FRY2 as bait to conduct another round of screens. We isolated six clones of HOS5, along with several other nuclear proteins (our unpublished data).

To map the interacting domain(s) of FRY2 and HOS5, we constructed full-length HOS5 in the pBD vector and full-length and various truncated FRY2 into the pAD vector. As shown in Figure 3H, yeast cells expressing constructs with the first dsRNA-binding domain (DRM) of FRY2 could grow on minus tryptophan/-leucine/-histidine/-adenine (-TLHA) medium, indicating that HOS5 interacts with the first DRM domain of FRY2, which is consistent with the notion that DRM can be either a dsRNA binding motif or a protein-protein interaction motif [33]. We also made deletion constructs of HOS5 to map its interaction domain(s) with FRY2. We found that cells containing constructs with the 3^rd^ and 4^th^ KH-domain could grow on -TLHA medium, whereas those lacking these two domains or only having parts of them could not grow, indicating that the region harboring the 3^rd^ and 4^th^ KH-domain of HOS5 is responsible for interacting with FRY2. Interestingly, the *hos5-1* mutation did not affect the HOS5-FRY2 interaction (Figure 3I). Furthermore, HOS5 interacted with itself through the second KH-domain and the *hos5-1* mutation did not affect HOS5 dimerization either (Figures 3J).

To search for additional proteins that may interact with HOS5, we performed co-immunoprecipitation (Co-IP) assays. Protein extracts from seedlings of the Col-0 (control) and the HOS5-FLAG-tagged lines were subjected to Co-IP with anti-FLAG M_2_ beads using standard protocols. The protein complexes bound with the beads were eluted with 3× FLAG peptide. The eluted proteins were then subjected to trypsin digestion and mass spectrometry analysis. We identified four peptide fragments that matched to an uncharacterized SR protein RS41 (Figure 3A). This protein belongs to the RS subfamily of SR splicing factors [17]. All 4 peptides were found to be in the RNA recognition motifs (RRM) at the N-terminus (Figure 3B). In Arabidopsis, there are 19 SR genes that are divided into 6 subgroups. Figure S6A shows the phylogenetic tree of these SR proteins. We found that the RS40 had the highest similarity with RS41, sharing 74.7% consensus sequence in the total protein length and 91.8% consensus sequence in the RRM. Interestingly, all 4 peptide fragments identified by mass spectrometry in RS41 also matched the sequence of RS40 except for one residue substitution (Figure S6B). It is therefore possible that RS40 may also be an interacting protein of HOS5.

To confirm the interaction between HOS5 and these putative splicing factors, constructs containing FLAG tag-fused HOS5 and Myc tag-fused RS40 and RS41 were made and expressed in pairs in tobacco (*N. benthamiana*) leaves by *Agrobacterium*-mediated leaf disc transformation. Co-IP was carried out by using anti-FLAG antibody for the pull down and normal IgG as a control. Samples were subjected to SDS-PAGE and western blot analysis. We found that both RS40 and RS41 could be pulled down by anti-FLAG antibody (Figures 3C and 3D), demonstrating that HOS5 indeed physically interacts with RS40 and RS41.

We next examined whether these proteins are co-localized in plant cells. Constructs with HOS5 fused with EGFP and RS40 and RS41 fused with mRFP were made, respectively. Two plasmid combinations, HOS5 with RS40 and HOS5 with RS41, were transformed into Arabidopsis protoplasts and visualized under a confocal microscope after the fusion proteins were transiently expressed for 16 hr. Figures 3E and 3F show that HOS5 co-localizes with RS40 and RS41 mostly in the nuclear speckles.

**Figure 4:**
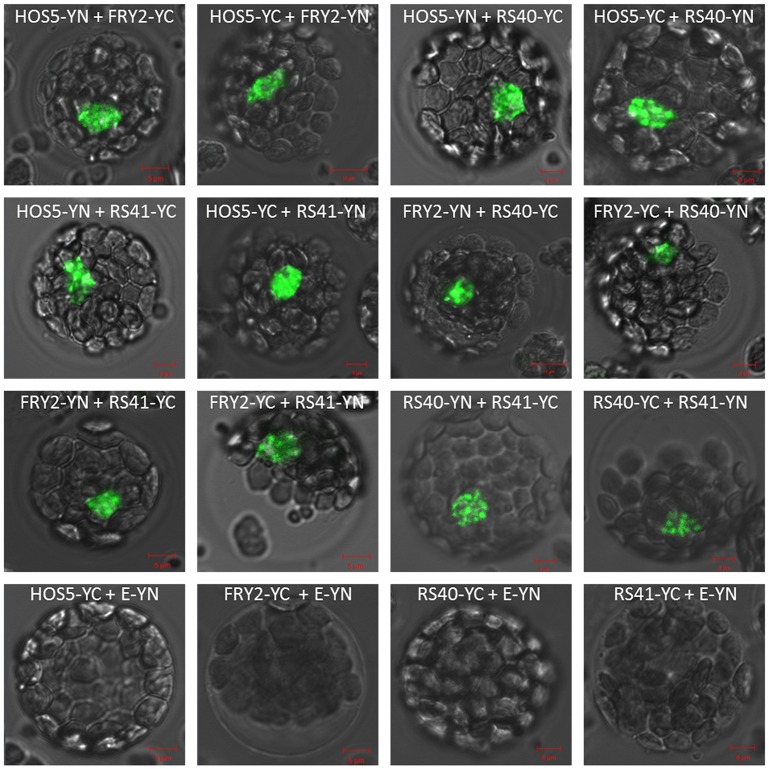
In vivo interactions between HOS5, FRY2 and the RS proteins in Arabidopsis. HOS5, FRY2, RS40 and RS41 were fused to EYFP-N or EYFP-C, respectively. E-YN and E-YC are empty vectors. The combinations of plasmids were transformed into Arabidopsis protoplast as indicated. Data for additional controls (YN-empty, YC-empty and interaction with the transcription factor HAP5C) were presented in Figure S4.

To verify that HOS5 interacts with the RS proteins and FRY2 in vivo, we performed bimolecular fluorescence complementation (BiFC) assays in Arabidopsis protoplasts. *HOS5*, *hos5-1*, *FRY2*, *RS40* and *RS41* were constructed to fuse either with N-EYFP or C-EYFP. HAP5C, a transcription factor, was used as negative control (Figure S4). As expected, HOS5 interacts with FRY2, RS40, and RS41 in vivo (Figure 4) but does not interact with HAP5C (Figure S4). Meanwhile, RS40 and RS41 interact with each other. Interestingly, FRY2 also interacts with RS40 and RS41, which indicates that HOS5-FRY2-RS40/41 may form a complex or part of a complex. Furthermore, BiFC also confirmed that HOS5 interacts with itself and may form dimers in vivo.

### Mutants in RS40 and RS41 had similar stress-sensitive phenotypes as *hos5*

Since HOS5 interacts with RS40 and RS41 as well as with FRY2 (Figures 3 and 4) and *fry2* mutants are similarly sensitive to stress like *hos5* (Xiong et al., 2002), we hypothesized that the null mutants of RS40 and RS41 may also have similar phenotypes as the *hos5* mutants. We obtained two T-DNA insertion lines, WiscDsLox382G12 (named as *rs40-1*) and SAIL-64-C03 (named as *rs41-1*) (Figure S6C). The expression levels of the two genes in these mutants were nearly undetectable by qPCR analysis (Figure S6D). These mutants were first tested for their sensitivity to ABA during seed germination. Figure S6E shows that compared with that of Col-0 or *hos5*-2, germination (radicle emergence) of *rs40-1* and *rs41-1* seeds was more strongly inhibited by ABA, particularly in the early stages. After germination, all cotyledons of Col-0 seedlings became green, whereas all cotyledons of *hos5-2* were yellow. On the other hand, approximately 50% of the cotyledons of *rs40-1* and *rs41-1* were yellow (Figure S6F). We also checked the salt stress sensitivity among these mutants. *hos5-2*, *rs40-1* and *rs41-1* were all more sensitive to the inhibition of root elongation by salt stress (Figure S6G). Furthermore, RS40 and RS41 had similar expression patterns and their expression levels were slightly enhanced by ABA and salt stress treatments (Figures S6H and S6I).

### Mutation in HOS5 and FRY2 results in intron retention during pre-mRNA processing

**Figure 5:**
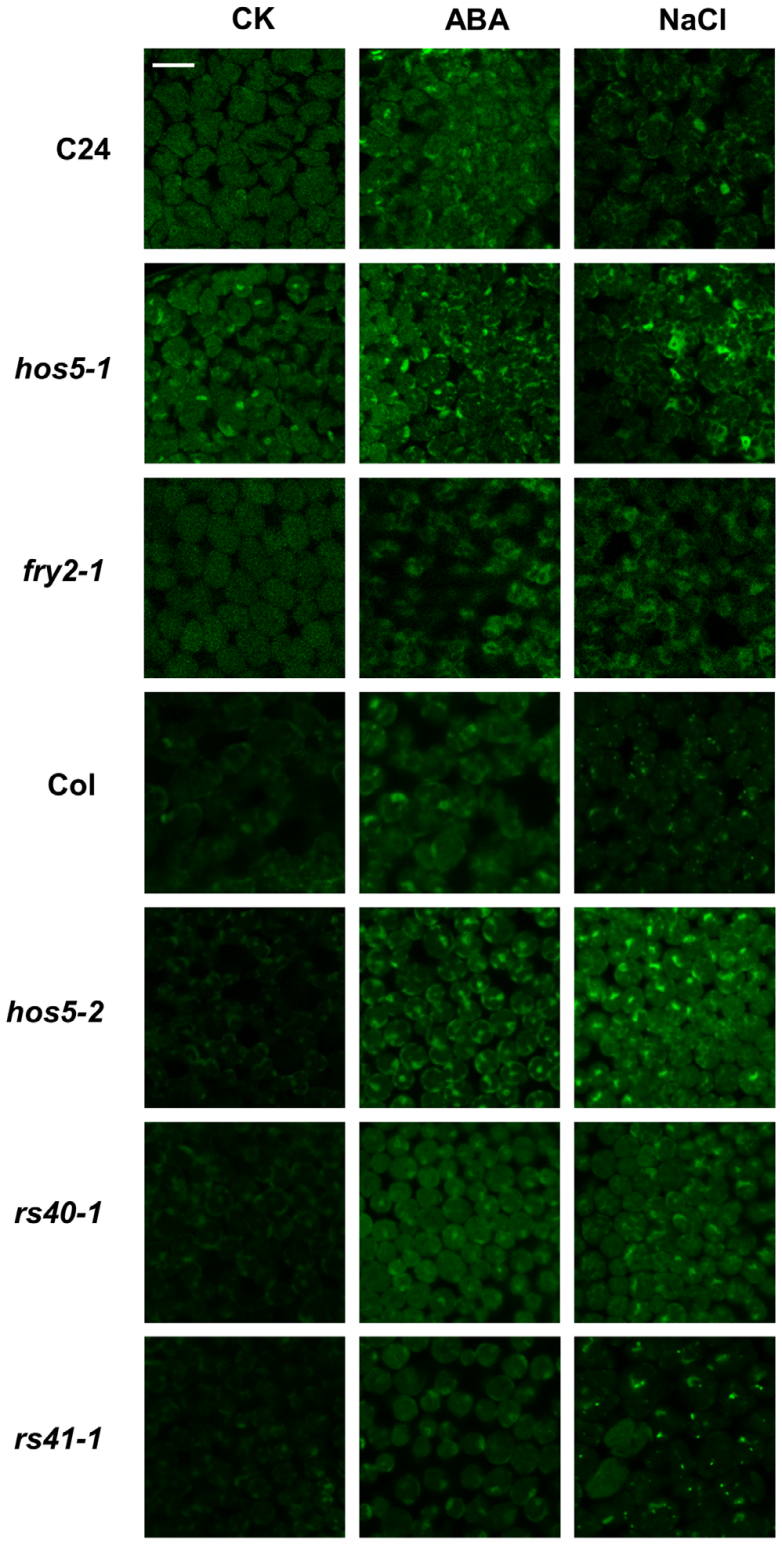
Impairment of mRNA export in *hos5*, *fry2* and *rs* mutants. Seedlings of C24, *hos5*-*1*, *fry2*-*1*, Col-0, *hos5-2*, *rs40-1* and *rs41-1* grown at 22°C for 2 weeks were treated with H_2_O (CK), 100 µM ABA or 300 mM NaCl for 3 hr. The samples were fixed by formaldehyde and in situ hybridization was performed with Alexa Fluor 488-labelled oligo-dT probe. Hybridization signals were visualized with a confocal microscope. White scale bar, 50 µm.

Since HOS5 co-localized with CypRS64 in nuclear speckles and interacted with two putative splicing factors, we posited that HOS5 might have a role in pre-mRNA splicing. It is known that abnormally processed mRNA may not be efficiently exported to the cytosol [34], [35]. We therefore first examined whether there was an accumulation of mRNA in the nuclei of *hos5*, *fry2-1* and the two *rs* mutants. Leaf samples from seedlings without or with NaCl treatment were fixed by formaldehyde and hybridized with an oligo-dT probe labeled with Alexa Fluor 488. As shown in Figure 5, there was only a slight difference in the Fluor 488 signal strength between the wild type and *hos5-1* under normal conditions. When treated with NaCl or ABA, both the wild type and the mutants had an increased accumulation of polyA RNA in the nuclei. Nonetheless, *hos5-1* and to a lesser extent, *fry2-1*, accumulated more polyA mRNA in the nuclei than did the wild type, especially under NaCl treatment (Figure 5). Among these mutants, the defect was particularly evident in *hos5* whereas *rs40* and *rs41* were much less affected.

To uncover the possible role of HOS5 and its interacting partners in pre-mRNA processing including splicing, we performed RNA sequencing (RNA-seq) using the Illumina Hi-Seq platform to examine the global pre-mRNA splicing in the wild type and mutants under salt stress. These mutants included the respective single mutants as well as *hos5-1 fry2-1* and *rs40 rs41* double mutants. We chose the salt stress treatment because we found that salt stress gave rise to the most distinguishable difference in the molecular phenotypes (e.g., the *LUC* gene expression and the nuclear accumulation of mRNAs) between the wild type and *hos5-1* mutant. We generated 359 million single-end/paired-end reads (101 bp in length), respectively. On average, about 90% of these reads could be unambiguously aligned to the TAIR10 reference genome sequence (fragments per kilobase of exons per million mapped reads - FPKM>0.85). Comparison of the mapped reads against the gene model (version TAIR10) revealed that ∼95% of the reads mapped to the exonic regions, whereas only ∼3% mapped to intergenic regions. Plotting the coverage of reads along each transcribed unit exhibited a uniform distribution, with no obvious 3′/5′ bias, which reflects a high quality of the cDNA library (data not shown). Furthermore, assessing the sequencing saturation showed that as more reads were obtained, the number of newly discovered genes plateaued (Figure S7A), suggesting that extensive coverage was achieved, which can also be supported by plotting the read coverage along each chromosome that showed extensive transcriptional activity in the genome (data not shown).

Compared with the wild type, in *hos5-1* mutants, 57 intron retention events (p<0.01, 4 times up-regulation) were represented. In *fry2-1*, *hos5-1 fry2-1* double mutants and *rs40-1 rs41-1* double mutants, the intron retention events were 247, 586, and 47, respectively. Interestingly, Gene Ontology (GO) analysis of the genes undergoing aberrant splicing in the above mutants revealed a striking enrichment in the response-to-abiotic-stress categories (Figure S7B). Notably, a number of known stress-tolerance genes were found to be defective in splicing in both *hos5-1* and *fry2-1* (Tables S2, S3 and S4). These include, for example, the Ca^2+^/H^+^ exchanger CAX1 (AT2G38170), which is activated by the SOS2 kinase and links Ca^2+^ with salt tolerance [36], CIPK3 (AT2G26980), which regulates ABA and stress response during seed germination [37], *RCI2A*, a cold-inducible gene that also enhances salt tolerance [38], and Rap2.6L, a transcription factor that activates the expression of stress-responsive genes [39]. The impaired splicing of these or similar genes may underlie the increased sensitivity to stress in these mutants. Overall, there are many common intron retention events among *hos5-1*, *fry2-1*, and *hos5-1 fry2-1* double mutants. For instance, half of alternative splicing events in *hos5-1* were also found in the *fry2-1* mutant, which is statistically very significant compared with those from random sampling of annotated introns (Fisher's Exact test, P value<0.001) (Figure S8).

**Figure 6:**
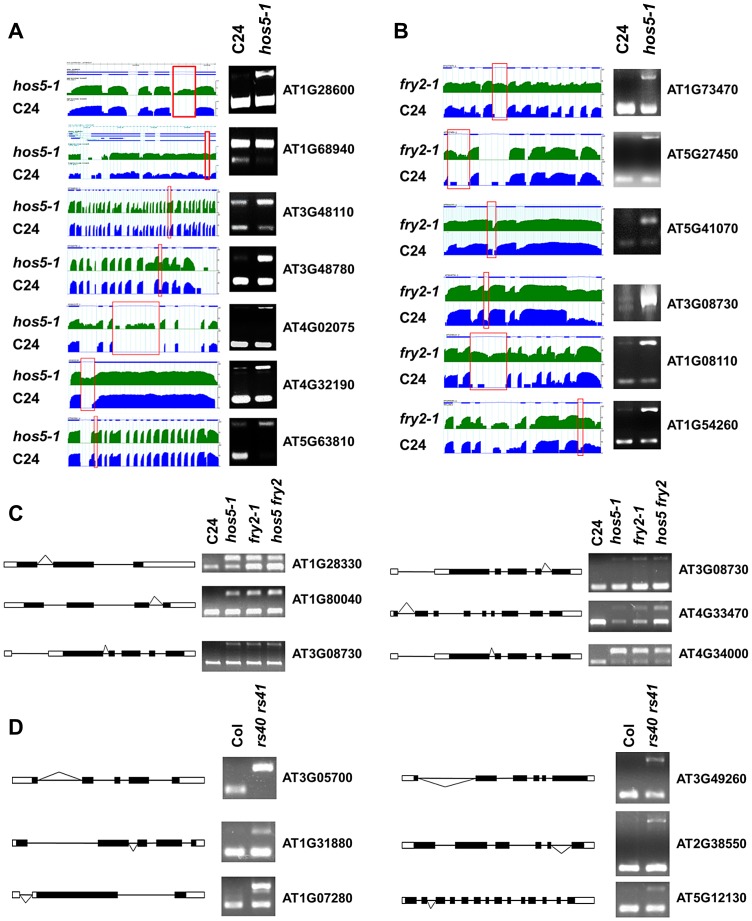
HOS5, FRY2 and RS40/41 are required for pre-mRNA splicing. (A) and (B) Selected intron retention events in *hos5-1* (A) and *fry2-1* (B). Transcripts with splicing defects detected by RNA-seq are shown (Left panels). For each gene, annotated gene structures are presented (Top), with thick lines representing exons and thin lines representing introns. Wiggle plots representing the normalized read coverage in a logarithmic scale (log10) are shown in green for *hos5-1* mutant (middle) and in blue for C24 (Bottom). The red frames indicate the validated introns. Validation of the intron retention events in these 6 genes by RT-PCR are shown (right panels). The upper and lower bands represent the unspliced and spliced forms, respectively. (C) The common target of intron retention events in *hos5-1*, *fry2-1* and *hos5-1 fry2-1* double (*hos5 fry2*) mutants. The paradigms of six representative genes were shown and the checked introns were marked by fold lines. (D) Splicing defects in the *rs40-1 rs41-1* double (*rs40 rs41*) mutant. The paradigms of six representative genes were shown and the checked introns were marked by fold lines. The RT-PCR validation was performed and the upper and lower bands represent the unspliced and spliced forms, respectively.

We selected six genes with intron retention events and validated them by RT-PCR using intron-flanking primers. Figures 6A and 6B showed intron retention validation results for *hos5-1* and *fry2-1*, respectively. Figure 6C presented the validation for intron retention events common to *hos5-1*, *fry2-1*, and *hos5-1 fry2-1* double mutants. Validation for intron retention in *rs40-1 rs41-1* double mutants was presented in Figure 6D. These data confirmed that the corresponding retained introns were detected in the respective mutants, whereas they were not or were only weakly expressed in the wild type. For some other genes (e.g., At5g63810 and At3g08730), the selected introns were partly spliced correctly in the wild type seedlings but they could not correctly spliced in the mutant seedlings at all (Figures 6A and 6B), suggesting that HOS5 and FRY2 also enhance the splicing efficiency of these weak introns. These data clearly demonstrate that HOS5 and its interacting components are involved in pre-mRNA splicing.

### FRY2 is required for correct localization of HOS5

**Figure 7:**
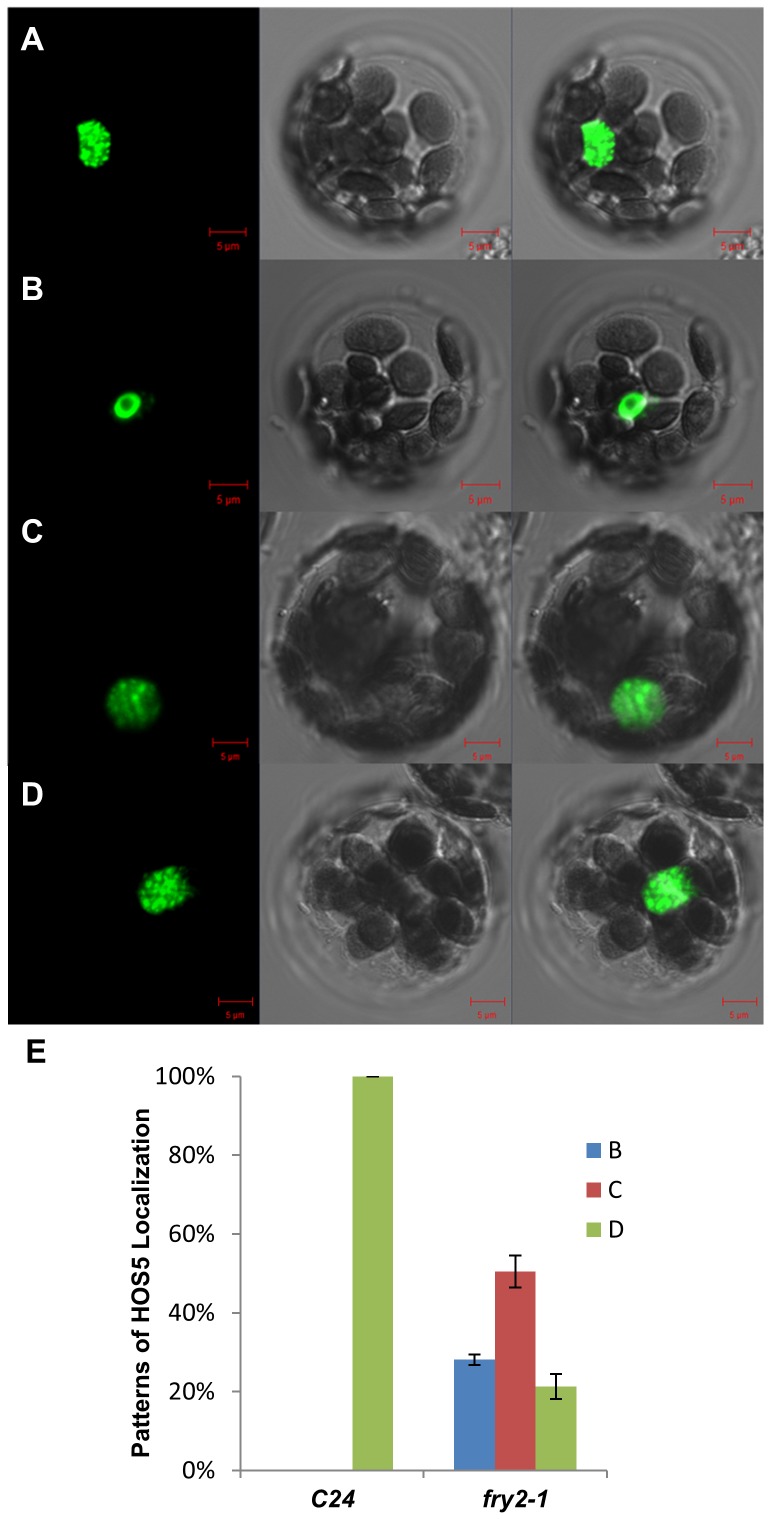
FRY2 is required for the correct localization of HOS5. A plasmid of HOS5-GFP was transferred into Arabidopsis C24 (A) and *fry2-1* (B, C, D) protoplasts and transiently expressed. Images were taken 16 h after the transformation with a confocal microscope. Left, GFP signal; middle, bright field images; right, merged images of the GFP and bright field signals. Scale bars represent 5 µm. (A) The representative image showing the localization of HOS5 in C24. (B) to (D) Representative patterns of HOS5 localization in *fry2-1*. (E) Percentage of different localization patterns of HOS5 in C24 and the *fry2*-1 mutant. Pattern B (blue bar), nucleoplasm localization (excluded from the nucleolus) (as shown in B); Pattern C (brown bar), diffused localization throughout the nucleus with accumulation in a few nuclear speckles (as shown in C); Pattern D (green bars), nuclear speckle localization (as shown in D). The Pattern D localization is the same as in the wild type (A). Data are average and standard derivations from three replicates each with 50 to 60 protoplasts counted per genotype.

The specific localization of HOS5 and FRY2 proteins in the nucleus prompted us to ask how these proteins are recruited to their particular subnuclear location, namely, nuclear speckles. We transiently expressed the respective GFP-tagged proteins in leaf protoplasts prepared from the wild type, *fry2*-*1* or *hos5-1* mutant seedlings. Whereas the localization of the FRY2 protein in the *hos5-1* mutant was not altered (data not shown), we found that most of the HOS5 protein was mislocalized in the *fry2-1* mutant. As shown in Figure 7, in contrast to its exclusive localization in the nuclear speckles in the wild type, HOS5 was mislocalized in nearly 80% of the protoplast cells of the *fry2-1* mutant. These included the nucleoplasm localization (but absent from the nucleolus) in 28% of the protoplast cells and mostly diffused localization in the nucleus with accumulation in a few nuclear speckles in 51% of the protoplast cells of the *fry2-1* mutant. Only in 21% of the protoplast cells of the *fry2-1* mutant was the HOS5 protein correctly localized in nuclear speckles. Overall, these data reveal that FRY2 is essential for the recruitment of HOS5 to nuclear speckles and presumably this will also affect HOS5's role in pre-mRNA processing.

## Discussion

HOS5 is a negative regulator of osmotic stress-responsive gene expression [23] and *hos5* mutants are more sensitive to osmotic stress (Figures S1 and S3). In this study, we identified HOS5 as a KH-domain protein. The K homology (KH) domain was originally identified in the heterogeneous nuclear ribonucleoprotein K (hnRNP K) of humans but it is also found in various proteins in archaea, bacteria and other eukaryotes [40], [41]. The KH domain is a RNA-binding domain normally found in multiple copies [42], [43], although single KH-domain proteins also exist. In Arabidopsis, there are 26 KH-domain proteins [44]. Since KH-domain proteins play critical roles in cell differentiation and development in metazoans, it is expected that these proteins may also function in plant development [45]. However, very few KH-domain proteins have been characterized in plants so far. One KH-domain protein, HUA ENHANCER 4 (HEN4), was found to interact with HUA1 in nuclear speckles to regulate flower development [46]. FLK, another KH-domain protein, controls flowering time and can be regulated by alternative splicing in the *atprmt5* mutant [19], [47]–[49]. Our discovery that HOS5 functions in plant stress resistance reveals a new role of KH-domain proteins in plants. That HOS5 was recently identified as required for heat stress tolerance [24] further strengthens this notion.

The *hos5-1* mutant has a mutation in the conserved glycine residue in the second KH domain, which likely affects the ability of HOS5 to bind its RNA targets. We attempted to isolate the RNA targets, but perhaps due to their low abundance, we could not identify with confidence the RNA species that are specifically bound by HOS5. However, through the identification of protein partners, we were able to uncover the molecular functions and modes of action of HOS5. In yeast two-hybrid screens, we identified the Pol II CTD phosphatase-like FRY2/CPL1 as an interacting partner. It has been demonstrated that FRY2/CPL1 can dephosphorylate Ser-5 but not Ser-2 of phosphorylated CTD [31]. Mutations of FRY2/CPL1 confer an increased expression of stress-responsive genes, a phenotype similar to that of *hos5-1* [30], [32], although the expression level of *RD29A::LUC* in *fry2-1* is slightly higher than that in *hos5-1*(Figure S5). We generated homozygous *hos5-1 fry2-1* double mutants and found that the double mutants had similar *RD29A::LUC* expression as *fry2-1* both under normal and salt stress conditions (Figure S5). This non-additive phenotype seen in the double mutant indicates that HOS5 and FRY2 function in the same or an overlapping pathway and that FRY2 may be epistatic to HOS5, consistent with the fact that they interact with each other. One interesting observation is that the interaction between these two proteins was mediated by the first DRM domain of FRY2 and the third and fourth KH domains of HOS5 (Figure 3H and 3I). These findings are consistent with the notion that both DRM and KH domains can also serve as protein-protein interaction domains in addition to binding RNA [33], [50]–[52]. Currently, it is unclear whether RNA binding would cooperate or compete with protein binding, but the *hos5-1* mutation in the KH2 domain does not affect the interaction between HOS5 and FRY2 (Figure 3I) or the interaction with itself, suggesting that the *hos5-1* mutation may affect RNA binding only.

To isolate additional proteins that interact with HOS5, we conducted Co-IP assays. This resulted in the identification of two uncharacterized SR proteins, RS40 and RS41, as HOS5 interacting proteins. It is interesting to note that the yeast two hybrid and Co-IP did not identify the same set of proteins, suggesting that the interaction strength may be different in yeast and in plants. Alternatively, different associated factors or the methodology itself may only be able to uncover certain interactions in these two systems. We therefore wanted to verify whether these proteins interact in plants.

In BiFC assays, HOS5 clearly interacted with FRY2/CPL1 and RS40/41 in vivo. Furthermore, FRY2 also interacted with RS40 and RS41. Interestingly, these interactions occurred predominately in nuclear speckles, although weaker interactions were also observed in the nucleoplasm (Figure 4). In contrast, HOS5 did not interact with another nucleus-localized transcription factor, HAP5C (Figure S4). These results suggest that these three proteins may form a complex or are part of a complex, presumably involved in pre-mRNA splicing.

To test this hypothesis, we conducted RNA-seq analysis of these mutants under both control and salt stress conditions. While we did not observe significant changes in splicing patterns under the control conditions (data not shown), we indeed detected clear impairments in pre-mRNA splicing in these mutants under salt stress conditions. We found that intron retention is common among the mutants, with the retention in *hos5-1 fry2-1* double mutant most dramatic, followed by *fry2-1* and *hos5* as well as *rs40 rs41*. These distinct impacts on pre-mRNA splicing are consistent with the perception that FRY2/CPL1 may have a broader or general role in regulating pre-mRNA processing than either HOS5 or the two RS proteins. The synergistic effect of FRY2 and HOS5 in regulating pre-mRNA splicing also implies that these two proteins may cooperatively regulate other pre-mRNA processing such as 5′ capping or transcript stability that may directly or indirectly enhance the splicing process.

The fact that both *hos5* and *fry2* mutants have defects in pre-mRNA splicing and that HOS5 and FRY2/CPL1 both interact with the splicing factors RS40 and RS41 in nuclear speckles is consistent with the idea that these proteins are involved in splicing of pre-mRNA. Nuclear speckles are interchromatin domains where many splicing factors, including snRNP and SR proteins, are stored and modified to be supplied to active transcription sites and thus are closely associated with gene transcription [53], a process that requires co-transcriptional recruitment of many pre-mRNA processing proteins. We attempted several times to detect whether HOS5 and FRY2 interaction involves the dephosphorylation of HOS5 by FRY2, yet due to unknown reasons, we could not obtain reproducible results. Nonetheless, we did find that FRY2 is required for the recruitment of HOS5 to nuclear speckles (Figure 7).

Previously, phosphorylated CTD was considered important for the recruitment of the spliceosome [54]. SR proteins were also found to interact with CTD and were suggested to be recruited to RNAP II to couple transcription to pre-mRNA processing [55]. Yet ChIP assay results and other evidence do not suggest ‘piggybacking’ of spliceosome assembly on CTD of RNAP II and rather suggest the involvement of other components in recruitment of splicing factors [56]. FRY2/CPL1 may act as such a component and play particular roles in the recruitment of specific splicing factors for certain transcripts such as those that function in stress response or, presumably, miRNA precursors, because, as was recently demonstrated, FRY2/CPL1 affects the processing accuracy of certain miRNA [57]. Indeed, in the current study, we found that in the *fry2-1* mutant, HOS5 protein could not accurately be recruited to nuclear speckles as in the wild type plants (Figure 7). The ability of FRY2 to recruit HOS5 to specific subnuclear foci suggests that FRY2 may be involved in other aspects of pre-mRNA processing such as 5′-capping and mRNA stability control since these processes will also need the recruitment and modification of many protein factors. As a large protein, FRY2/CPL1 may be particularly suited for the recruitment of pre-mRNA processing factors for co-transcriptional mRNA processing. The relatedness of this alternative recruitment strategy to RNAP II CTD recruitment and the other functions of FRY2 and HOS5 in other pre-mRNA processing events will be the focus of future work.

## Materials and Methods

### Plant growth and stress treatments

Arabidopsis plants were grown under standard conditions and stress physiological analyses were performed as previously described [23].

### Genetic mapping

The *hos5*-1 mutant in the C24 background was crossed with the Columbia ecotype. The resulting F_1_ plants were allowed to self-pollinate. Mutants with strong luminescence under ABA and NaCl treatment were selected from the F_2_ population. Genomic DNA was extracted from each mutant F_2_ plants and used for mapping with simple sequence length polymorphism markers [58].

### Real-time quantitative PCR and gene expression

Total RNA was extracted from seedlings by using the Plant RNeasy Kit with DNaseI treatment (Qiagen, CA, USA). cDNAs were synthesized from total RNA by using Superscript III reverse transcriptase (Invitrogen). Real-time quantitative PCR was performed on the ABI 7900HT Fast Real-Time PCR System using *ACT2* as a control. Primers used in this study are presented in Table S1.

### Promoter analysis and protein co-localization

*HOS5* promoter (1.8 Kb) was amplified from genomic DNA and ligated to pENTR-D-TOPO. The promoter was cloned into the destination vector pMDC162 [59] and transgenic plants were generated by *Agrobacterium*-mediated flower dipping. For the β-glucuronidase (GUS) analysis, plant samples were stained in GUS staining buffer at 37°C overnight followed by destaining with 75% ethanol.

For co-localization assays, HOS5, CypRS64, FRY2, RS40 and RS41 were cloned into pENTR-D-TOPO and transferred to destination vectors containing EGFP or mRFP respectively by LR reaction. Plant seedlings and protoplasts expressing these fused proteins were imaged with an LSM 710 inverted confocal microscope (Carl Zeiss, Germany).

### Co-immunoprecipitation assay and protein mass spectrometry identification

Standard Co-IP protocol was performed. Briefly, 2 g of leaves of HOS5-FLAG plants were grounded and lysis buffer (100 mM KCl, 10 mM HEPES, 2 mM EGTA, 10 mM MgCl2, 10% Glycerol, 0.1% NP-40, 2 mM PMSF and 1× Protease Inhibitor Cocktail) was added to each sample. After centrifugation, the supernatant was transferred to a new tube. The samples were pre-cleaned by adding normal IgG-conjugated beads. The M2 beads were then added into the samples and incubated for 3 hr. Afterwards, the beads were washed 5 times with the lysis buffer and twice with TBS buffer. Proteins were eluted by adding 3×FLAG solution and were then subjected to SDS-PAGE and in-gel digested for mass spectrometry analysis.

### Yeast two-hybrid assays

Yeast transformation was performed according to the manufacturer's instructions (Clontech, CA). To map the interacting domain, FRY2 and HOS5 deletion constructs were made from both the N- and C-termini. These deletion mutants were then tested for interaction by co-transforming them into yeast strain AH109 with the corresponding full-length partner (FRY2 in pGAD or HOS5 in pGBK) and plating on -tryptophan/-leucine (-TL, for transformation control) and -tryptophan/-leucine/-histidine/-adenine (-TLHA, for selection) media supplemented with X-α-Gal. For FRY2, seven deletion constructs were tested. For the HOS5, eight deletion constructs and the hos5-1 mutant were tested.

### Poly-A RNA in situ hybridization

Poly-A RNA in situ hybridization was performed as described [60].

### Next-generation sequencing, RNA-seq analysis and validation of intron retention

Using the TRIzol Reagent (15596-026, Invitrogen), total RNA was extracted from 12-day-old seedlings treated with 300 mM NaCl for 3 hr. Polyadenylated RNA was isolated using the Oligotex mRNA Midi Kit (70042, Qiagen). The RNA-seq libraries were constructed using an Illumina Whole Transcriptome Analysis Kit following the standard protocol (Illumina, HiSeq system) and sequenced on the HiSeq platform to generate high-quality paired-end reads. For RNA-seq data analysis, Arabidopsis genome sequences and annotated gene models were downloaded from TAIR10 (http://www.arabidopsis.org/). The raw reads were aligned to genome sequences using TopHat (2.0.7) [61], allowing up to two mismatches. The gene expression levels (FPKM) were calculated with Cufflinks (2.0.2) [62]. Fisher's Exact Tests in R (http://www.r-project.org/) was performed to identify differential representation of introns in wild type and mutants using read coverage from an intron and the corresponding exons. Introns with more than 10 times coverage and two-sided P values less than 0.01 were regarded as the intron retention events. Primers were designed according to RNA-seq results with one primer in a primer pair covering the exon-exon junction [19] and semi-quantitative RT-PCR was performed for validation.

## Supporting Information

Figure S1 The luminescence and stress phenotypes of the *hos5-1* mutant. (A) Luminescence of the wild-type (left) and *hos5-1* (right) seedlings without treatment. (B) Luminescence after 100 µM ABA treatment for 3 hr. (C) Luminescence after 300 mM NaCl treatment for 3 hr. (D) *LUC* expression levels under different conditions measured by Real-Time Quantitative RT-PCR. *ACTIN2* was used as an internal control. Error bars represent the standard deviations (*n* = 3). (E) Seed germination under different concentrations of ABA. Seeds were surface-sterilized and planted on ½ MS agar media supplemented with the indicated concentrations of ABA and incubated at 4°C for 3 days before being placed at 22°C for germination. Germination (radicle emergence) was scored 3 days later. Results are means and standard errors from three replicates with 100 seeds for each treatment. (F) ABA sensitivity of *hos5-1* mutant seedlings. Seven-day-old *hos5-1* and C24 seedlings were transferred from ½ MS-agar media to media containing the indicated concentrations of ABA. The photos were taken 2 weeks after the transfer. (G) Root growth of *hos5-1* under salt stress. Four-day-old seedlings of C24 and *hos5-1* were transferred from ½ MS agar plates to plates supplemented with 120 mM NaCl. Root elongation was measured 7 days after the transfer. Results are means and standard errors (*n* = 12). (H) Seed germination sensitivity to salt stress. Seeds were planted on ½ MS medium plates with 100 mM NaCl and vernalized at 4°C for 3 days before being placed at 22°C for germination. Germination was then scored daily for 6 consecutive days. (I) *hos5-1* seedlings were slightly more sensitive to salt stress than C24 seedlings. Seven-day-old C24 and *hos5-1* seedlings were transferred from ½ MS-agar medium plates to ½ MS-agar plates supplemented with 0, 60, or 120 mM NaCl. The photos were taken 3 weeks after the transfer.
(TIF)

Figure S2 HOS5 structure and sequence comparison with other homologous proteins. (A) 3D-structure of HOS5 predicted by Phyre2. KH domains are alpha helixes and KH1 through KH5 are in blue, green, yellow, brown and red, respectively. (B) The cladogram analysis of all multi-KH domain-containing proteins in Arabidopsis. (C) Alignment of the 2^nd^ KH domain in KH domain-containing proteins of Arabidopsis. The red star indicates the most conserved glycine residue in the 2^nd^ KH domain, which was mutated in *hos5-1*.
(TIF)

Figure S3 *hos5-2* has similar phenotypes as *hos5-1*. (A) Structure of the *HOS5* gene and the locations of *hos5-2* and *hos5-3* mutations. Black boxes, exons; white boxes, untranslated regions (UTRs); lines, introns; star, *hos5-1* mutation site; black triangles, T-DNA insertion sites. (B) RT-PCR analysis was performed to check the expression level of *HOS5* in Col-0, *hos5-2* and *hos5-3* mutants. *ACTIN 2* was used as control. This experiment was repeated 3 times and the same result was obtained. (C) The phenotype of *hos5-2* under salt stress. Four-day-old seedlings of Col-0 and *hos5-2* were transferred on to vertical ½ MS agar plates without (left panel) or with (right panel) 120 mM NaCl. The pictures were taken 7 days after the transfer. (D) Root elongation of Col-0 and *hos5-2* under salt stress as shown in (C). Results are means and standard errors (n = 12). (E) Seed germination and early seedling development under ABA treatment. Seeds of Col-0 and *hos5-2* were surfaced sterilized and planted on ½ MS agar plates with 2.0 µM ABA. After 3-day vernalization, the plates were placed at 22°C for germination and growth. The picture was taken 7 days after the plate being incubated at the room temperature. (F) Relative root elongation of *hos5-2*. Four-day-old seedlings of Col-0 and *hos5-2* were transferred to ½ MS agar plates with 15 µM ABA. The root elongation was measured 7 days after the transfer. Results are means and standard errors (n = 12). Blue, Col-0; green, *hos5-2*.
(TIF)

Figure S4 Negative controls of the BiFC assays. HOS5, FRY2, RS40 and RS41 were respectively fused to EYFP-N. HAP5C, a transcription factor used as negative control, was fused to EYFP-C. E-YN and E-YC are empty vectors. The combinations of plasmids were transformed into Arabidopsis protoplasts as indicated. Shown are merged bright field and fluorescence images (no fluorescence signals were detected).
(TIF)

Figure S5 *RD29A-LUC* expression in *hos5-1 fry2-1* double mutants. (A) Luminescence images of C24, *hos5-1, fry2-1* and *hos5-1 fry2-1* double mutants without (CK) or with 300 mM NaCl treatment for 3 hr. T2–3, T2–6 and T2–22 are 3 lines of the double mutant. (B) Quantification of luminescence intensity in (A). Error bars represent standard deviation (*n* = 15). Blue, control treatment (CK); orange, NaCl treatment.
(TIF)

Figure S6 RS40 and RS41 sequence characters and the stress phenotypes of their mutants. (A) The cladogram analysis of all SR splicing factor proteins in Arabidopsis. RS40 and RS41 are clustered together as indicated in a red rectangle. (B) Sequence alignment of RS40 and RS41. The red lines indicate the peptide fragments identified by mass spectrometry. All 4 fragments are in the RRM motif. (C) Schematic structure of the *RS40* and *RS41* genes. Black boxes, exons; white boxes, UTRs; lines, introns; black triangles, T-DNA insertion sites. (D) Real-time qPCR analysis of the expression levels of *RS40* and *RS41* in Col-0, *rs40-1* and *rs41-1* mutants, respectively. *ACTIN 2* was used as a control. The experiment was repeated 3 times and similar results were obtained each time. (E) Seeds germination on ½ MS agar plates containing 2.0 µM ABA. Seeds were surface sterilized and incubated at 4°C for 3 days before being placed at 22°C for germination. The germination rates were scored daily for 7 consecutive days. Results are means and standard errors (*n* = 3). (F) Sensitivity of RS40 and RS41 seed germination to ABA treatment. The wild-type and mutant seeds were allowed to germinate and grow on 2.0 µM ABA plates. The picture was taken 7 days after germination. (G) Four-day-old seedlings of Col-0, *hos5-2*, *rs40-1* and *rs41-1* were transferred from ½ MS agar plates to ½ MS agar plates supplemented with 120 mM NaCl. Root elongation was measured. Results are means and standard errors (*n* = 12). Blue, Col-0; orange, *hos5-2*; green, *rs40-1*; purple, *rs41-1*. (H) and (I), Expression of the *RS40* and *RS41* genes under stress condition. *ACT2* was used as an internal control in qPCR assays. Error bars represent the standard deviations (*n* = 3).
(TIF)

Figure S7 The properties of *hos5-1* RNA-seq data and Gene Ontology of splicing-defective genes. (A) Randomly sampled reads were plotted against the mapped genes for the wild type and mutants. x-Axis shows the number of the mapped reads and y-axis displays the number of the expressed genes. (B) Gene Ontology of the genes with splicing defects in mutants.
(TIF)

Figure S8 A Venn diagram showing the number of genes with intron retention common among different mutants. *hos5*, the *hos5-1* mutant; *fry2*, the *fry2-1* mutant; *hos5/fry2*, the *hos5-1 fry2-1* double mutant. The list of genes with splicing defects in these mutants can be found in Table S2 to S4.
(TIF)

Table S1 List of primers used in this study.
(XLSX)

Table S2 List of genes with intron retention in the *hos5-1* mutant.
(XLSX)

Table S3 List of genes with intron retention in the *fry2-*1 mutant.
(XLSX)

Table S4 List of genes with intron retention in the *hos5-1 fry2-1* double mutant.
(XLSX)
