# Supplementary figures and images for: A KH-Domain RNA-Binding Protein Interacts with FIERY2/CTD Phosphatase-Like 1 and Splicing Factors and Is Important for Pre-mRNA Splicing in Arabidopsis
Source: PLoS Genet. 2013 Oct 17;9(10):e1003875. doi: 10.1371/journal.pgen.1003875 (PMC3798263; doi:10.1371/journal.pgen.1003875)

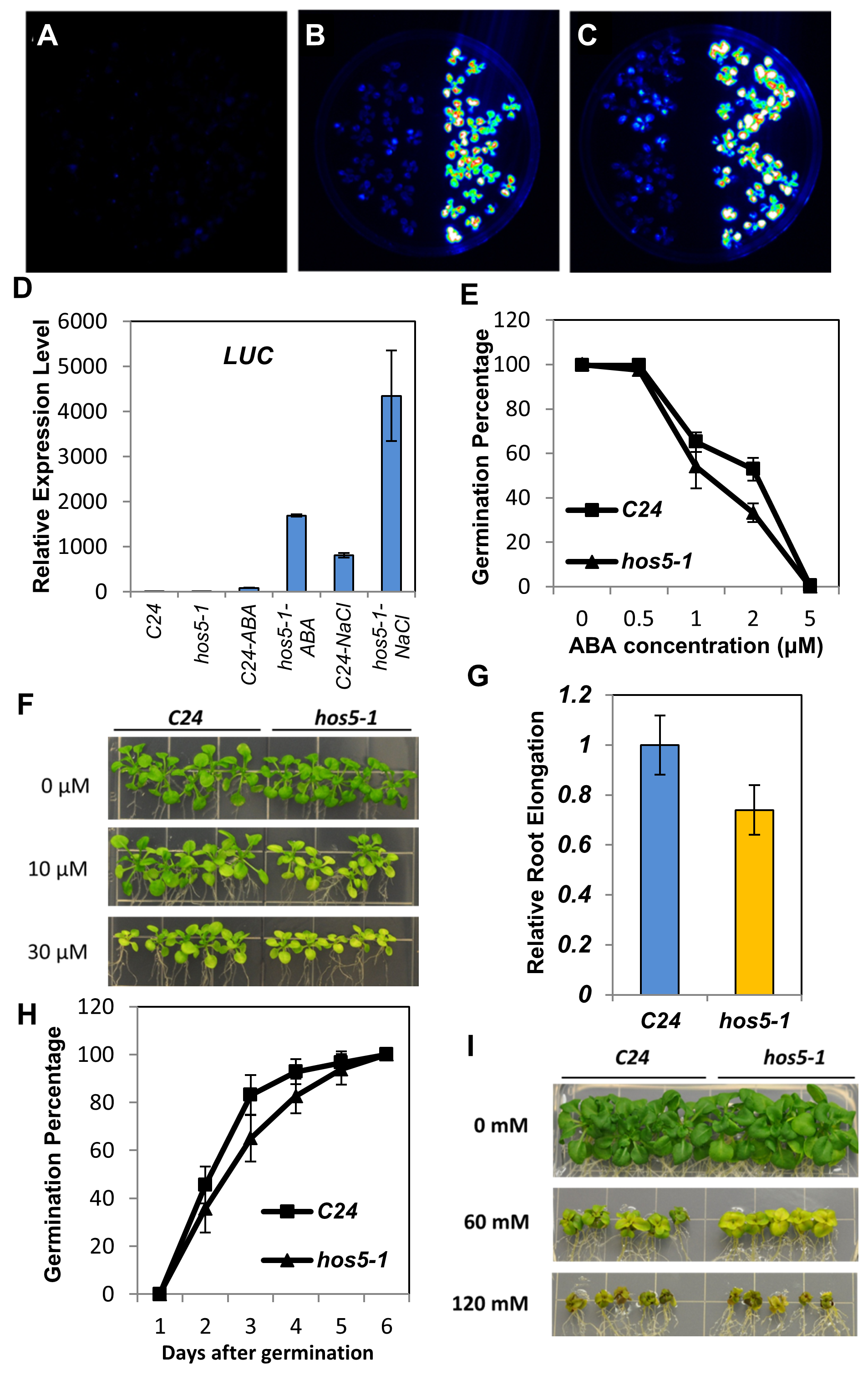

Supplement: Figure S1 — The luminescence and stress phenotypes of the hos5-1 mutant. (A) Luminescence of the wild-type (left) and hos5-1 (right) seedlings without treatment. (B) Luminescence after 100 µM ABA treatment for 3 hr. (C) Luminescence after 300 mM NaCl treatment for 3 hr. (D) LUC expression levels under different conditions measured by Real-Time Quantitative RT-PCR. ACTIN2 was used as an internal control. Error bars represent the standard deviations (n = 3). (E) Seed germination under different concentrations of ABA. Seeds were surface-sterilized and planted on ½ MS agar media supplemented with the indicated concentrations of ABA and incubated at 4°C for 3 days before being placed at 22°C for germination. Germination (radicle emergence) was scored 3 days later. Results are means and standard errors from three replicates with 100 seeds for each treatment. (F) ABA sensitivity of hos5-1 mutant seedlings. Seven-day-old hos5-1 and C24 seedlings were transferred from ½ MS-agar media to media containing the indicated concentrations of ABA. The photos were taken 2 weeks after the transfer. (G) Root growth of hos5-1 under salt stress. Four-day-old seedlings of C24 and hos5-1 were transferred from ½ MS agar plates to plates supplemented with 120 mM NaCl. Root elongation was measured 7 days after the transfer. Results are means and standard errors (n = 12). (H) Seed germination sensitivity to salt stress. Seeds were planted on ½ MS medium plates with 100 mM NaCl and vernalized at 4°C for 3 days before being placed at 22°C for germination. Germination was then scored daily for 6 consecutive days. (I) hos5-1 seedlings were slightly more sensitive to salt stress than C24 seedlings. Seven-day-old C24 and hos5-1 seedlings were transferred from ½ MS-agar medium plates to ½ MS-agar plates supplemented with 0, 60, or 120 mM NaCl. The photos were taken 3 weeks after the transfer. (TIF) [file pgen.1003875.s001.tif]

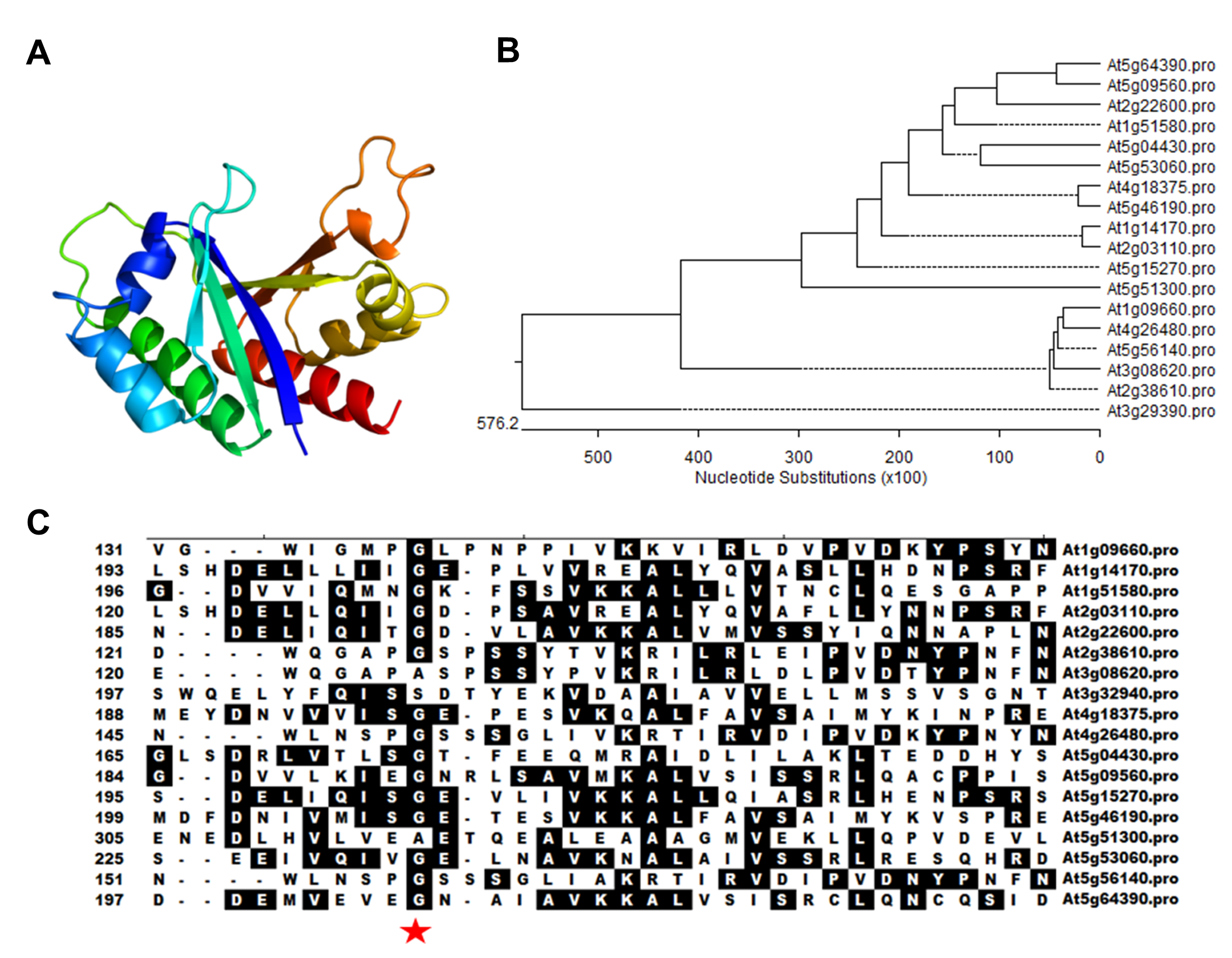

Supplement: Figure S2 — HOS5 structure and sequence comparison with other homologous proteins. (A) 3D-structure of HOS5 predicted by Phyre2. KH domains are alpha helixes and KH1 through KH5 are in blue, green, yellow, brown and red, respectively. (B) The cladogram analysis of all multi-KH domain-containing proteins in Arabidopsis. (C) Alignment of the 2nd KH domain in KH domain-containing proteins of Arabidopsis. The red star indicates the most conserved glycine residue in the 2nd KH domain, which was mutated in hos5-1. (TIF) [file pgen.1003875.s002.tif]

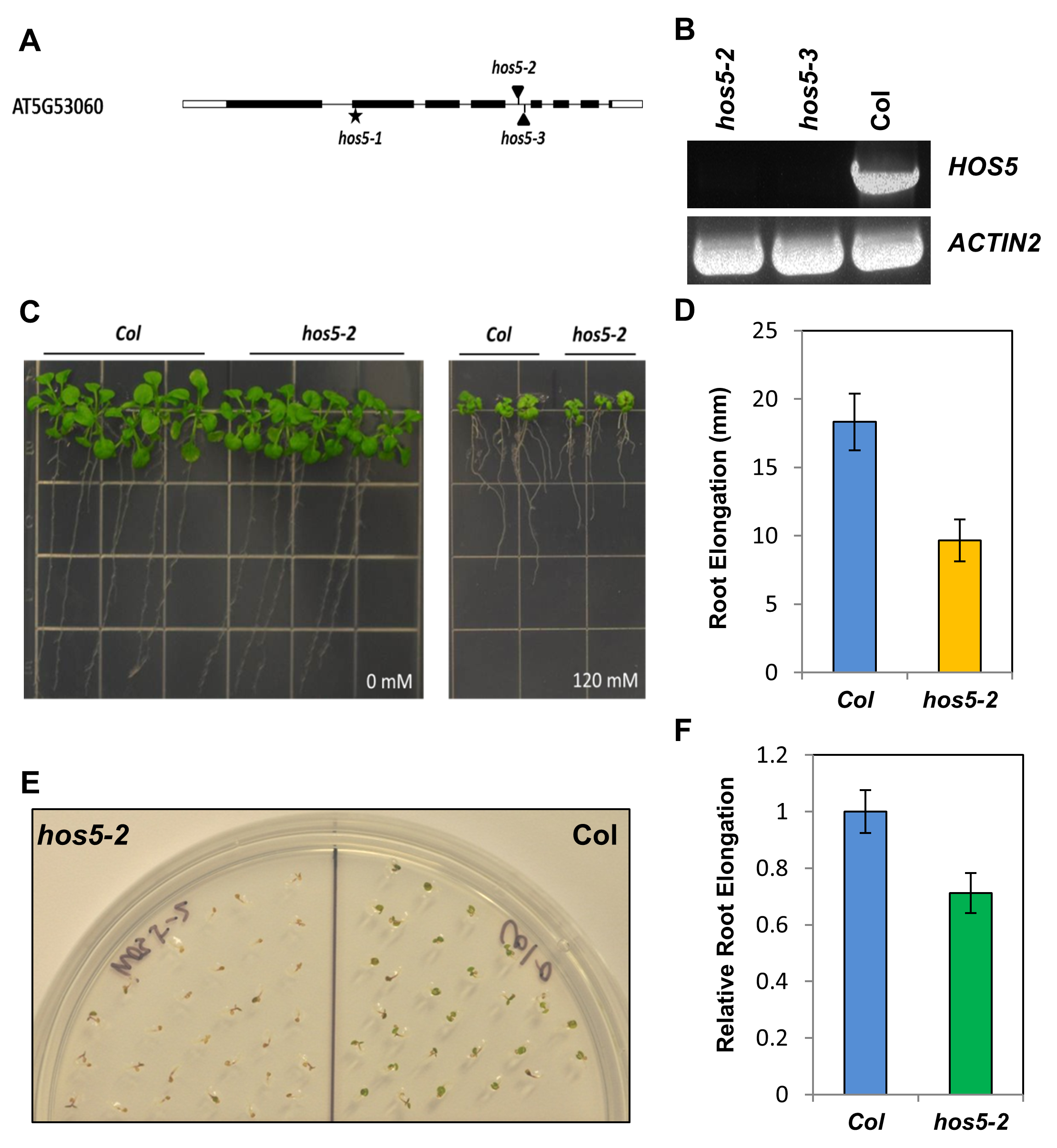

Supplement: Figure S3 — hos5-2 has similar phenotypes as hos5-1. (A) Structure of the HOS5 gene and the locations of hos5-2 and hos5-3 mutations. Black boxes, exons; white boxes, untranslated regions (UTRs); lines, introns; star, hos5-1 mutation site; black triangles, T-DNA insertion sites. (B) RT-PCR analysis was performed to check the expression level of HOS5 in Col-0, hos5-2 and hos5-3 mutants. ACTIN 2 was used as control. This experiment was repeated 3 times and the same result was obtained. (C) The phenotype of hos5-2 under salt stress. Four-day-old seedlings of Col-0 and hos5-2 were transferred on to vertical ½ MS agar plates without (left panel) or with (right panel) 120 mM NaCl. The pictures were taken 7 days after the transfer. (D) Root elongation of Col-0 and hos5-2 under salt stress as shown in (C). Results are means and standard errors (n = 12). (E) Seed germination and early seedling development under ABA treatment. Seeds of Col-0 and hos5-2 were surfaced sterilized and planted on ½ MS agar plates with 2.0 µM ABA. After 3-day vernalization, the plates were placed at 22°C for germination and growth. The picture was taken 7 days after the plate being incubated at the room temperature. (F) Relative root elongation of hos5-2. Four-day-old seedlings of Col-0 and hos5-2 were transferred to ½ MS agar plates with 15 µM ABA. The root elongation was measured 7 days after the transfer. Results are means and standard errors (n = 12). Blue, Col-0; green, hos5-2. (TIF) [file pgen.1003875.s003.tif]

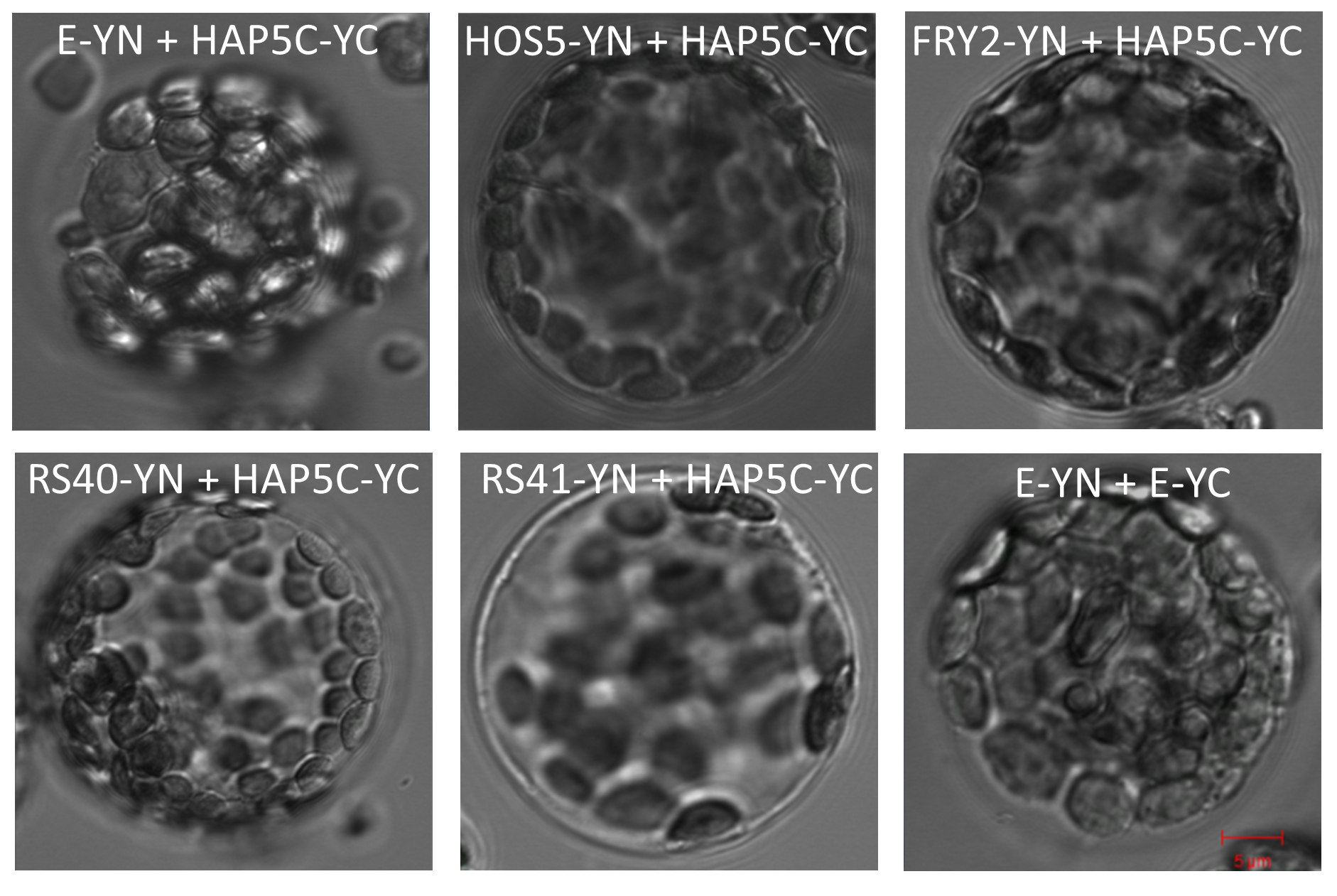

Supplement: Figure S4 — Negative controls of the BiFC assays. HOS5, FRY2, RS40 and RS41 were respectively fused to EYFP-N. HAP5C, a transcription factor used as negative control, was fused to EYFP-C. E-YN and E-YC are empty vectors. The combinations of plasmids were transformed into Arabidopsis protoplasts as indicated. Shown are merged bright field and fluorescence images (no fluorescence signals were detected). (TIF) [file pgen.1003875.s004.tif]

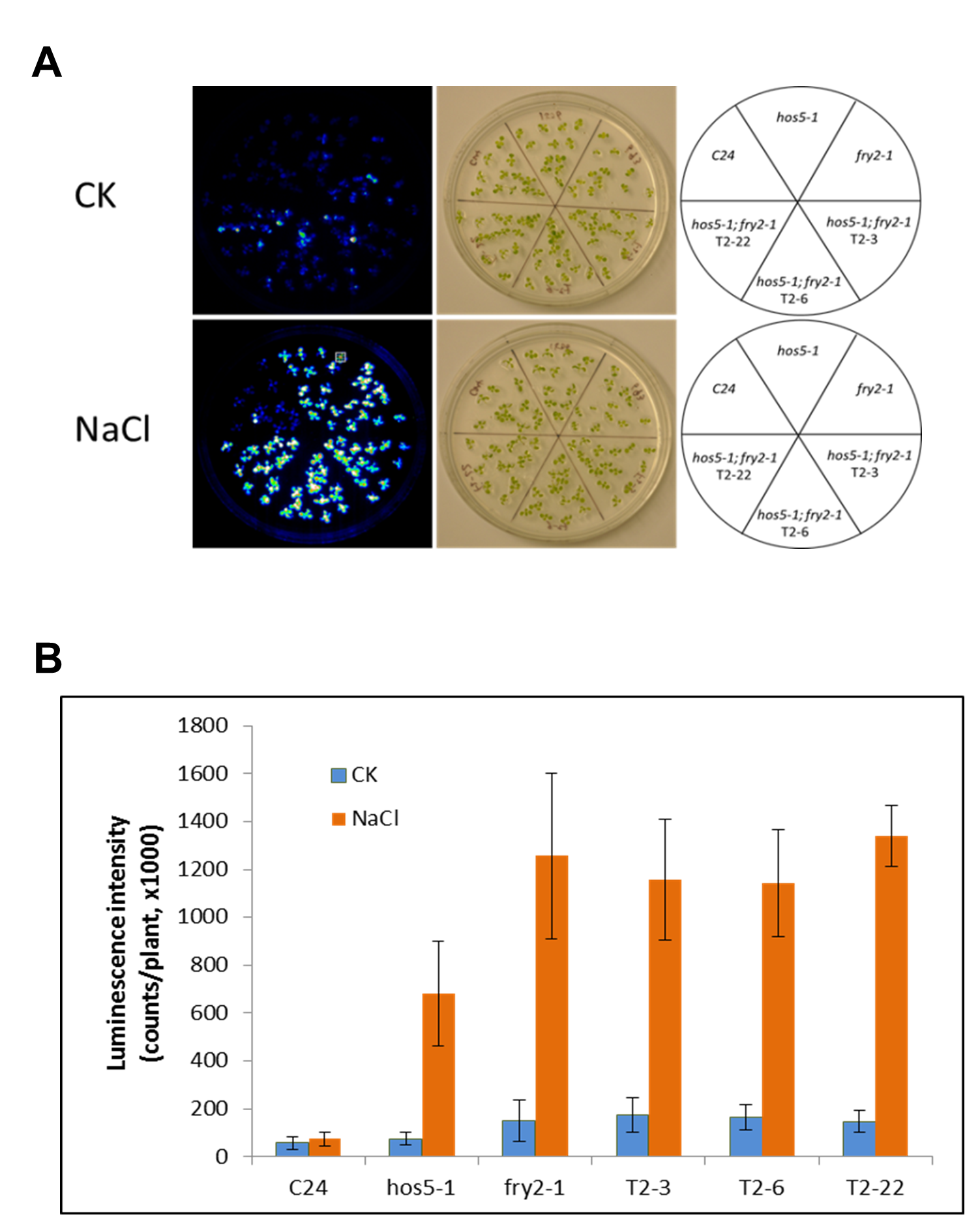

Supplement: Figure S5 — RD29A-LUC expression in hos5-1 fry2-1 double mutants. (A) Luminescence images of C24, hos5-1, fry2-1 and hos5-1 fry2-1 double mutants without (CK) or with 300 mM NaCl treatment for 3 hr. T2–3, T2–6 and T2–22 are 3 lines of the double mutant. (B) Quantification of luminescence intensity in (A). Error bars represent standard deviation (n = 15). Blue, control treatment (CK); orange, NaCl treatment. (TIF) [file pgen.1003875.s005.tif]

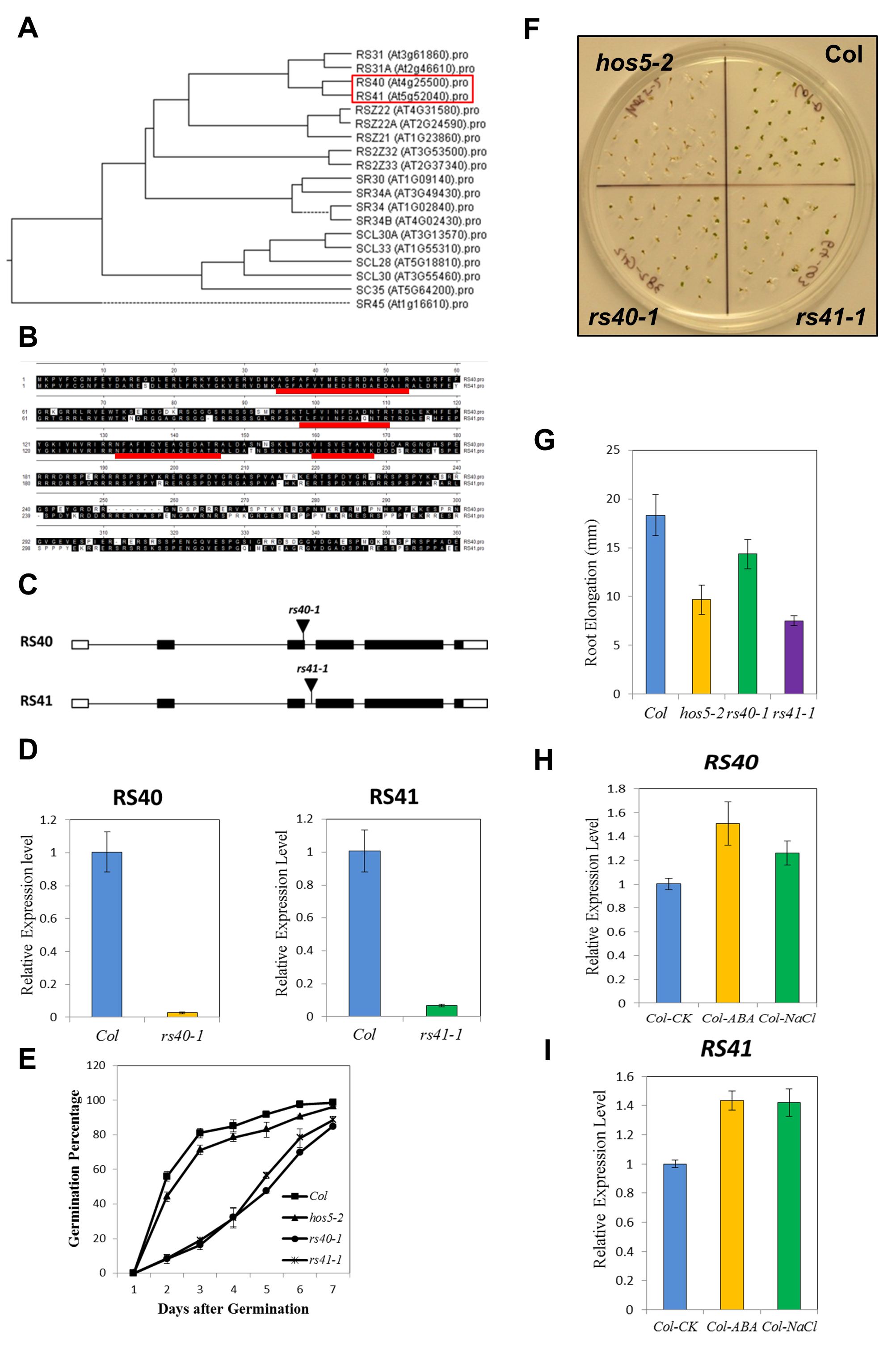

Supplement: Figure S6 — RS40 and RS41 sequence characters and the stress phenotypes of their mutants. (A) The cladogram analysis of all SR splicing factor proteins in Arabidopsis. RS40 and RS41 are clustered together as indicated in a red rectangle. (B) Sequence alignment of RS40 and RS41. The red lines indicate the peptide fragments identified by mass spectrometry. All 4 fragments are in the RRM motif. (C) Schematic structure of the RS40 and RS41 genes. Black boxes, exons; white boxes, UTRs; lines, introns; black triangles, T-DNA insertion sites. (D) Real-time qPCR analysis of the expression levels of RS40 and RS41 in Col-0, rs40-1 and rs41-1 mutants, respectively. ACTIN 2 was used as a control. The experiment was repeated 3 times and similar results were obtained each time. (E) Seeds germination on ½ MS agar plates containing 2.0 µM ABA. Seeds were surface sterilized and incubated at 4°C for 3 days before being placed at 22°C for germination. The germination rates were scored daily for 7 consecutive days. Results are means and standard errors (n = 3). (F) Sensitivity of RS40 and RS41 seed germination to ABA treatment. The wild-type and mutant seeds were allowed to germinate and grow on 2.0 µM ABA plates. The picture was taken 7 days after germination. (G) Four-day-old seedlings of Col-0, hos5-2, rs40-1 and rs41-1 were transferred from ½ MS agar plates to ½ MS agar plates supplemented with 120 mM NaCl. Root elongation was measured. Results are means and standard errors (n = 12). Blue, Col-0; orange, hos5-2; green, rs40-1; purple, rs41-1. (H) and (I), Expression of the RS40 and RS41 genes under stress condition. ACT2 was used as an internal control in qPCR assays. Error bars represent the standard deviations (n = 3). (TIF) [file pgen.1003875.s006.tif]

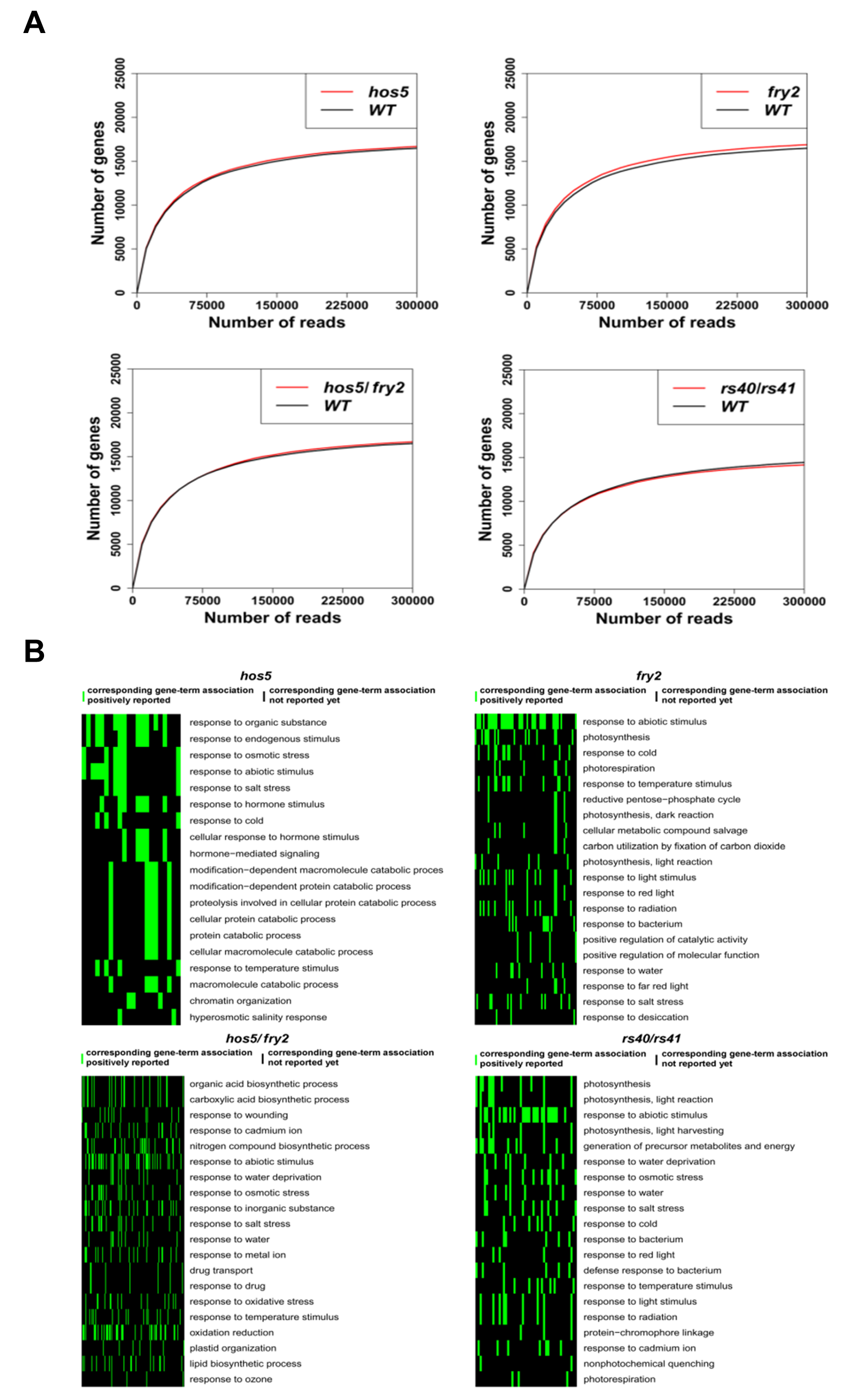

Supplement: Figure S7 — The properties of hos5-1 RNA-seq data and Gene Ontology of splicing-defective genes. (A) Randomly sampled reads were plotted against the mapped genes for the wild type and mutants. x-Axis shows the number of the mapped reads and y-axis displays the number of the expressed genes. (B) Gene Ontology of the genes with splicing defects in mutants. (TIF) [file pgen.1003875.s007.tif]

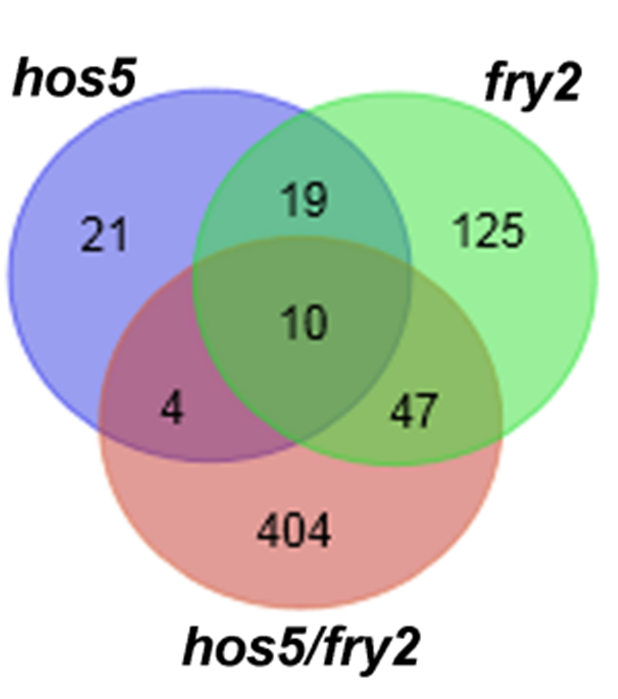

Supplement: Figure S8 — A Venn diagram showing the number of genes with intron retention common among different mutants. hos5, the hos5-1 mutant; fry2, the fry2-1 mutant; hos5/fry2, the hos5-1 fry2-1 double mutant. The list of genes with splicing defects in these mutants can be found in Table S2 to S4. (TIF) [file pgen.1003875.s008.tif]
